# Visual hallucinations induced by Ganzflicker and Ganzfeld differ in frequency, complexity, and content

**DOI:** 10.1038/s41598-024-52372-1

**Published:** 2024-01-29

**Authors:** Oris Shenyan, Matteo Lisi, John A. Greenwood, Jeremy I. Skipper, Tessa M. Dekker

**Affiliations:** 1 Experimental Psychology, Division of Psychology and Language Sciences, University College London, London, UK; 2 Institute of Ophthalmology, University College London, London, UK; 3 Department of Psychology, Royal Holloway University, London, UK

**Keywords:** Perception, Consciousness

## Abstract

Visual hallucinations can be phenomenologically divided into those of a simple or complex nature. Both simple and complex hallucinations can occur in pathological and non-pathological states, and can also be induced experimentally by visual stimulation or deprivation—for example using a high-frequency, eyes-open flicker (Ganzflicker) and perceptual deprivation (Ganzfeld). Here we leverage the differences in visual stimulation that these two techniques involve to investigate the role of bottom-up and top-down processes in shifting the complexity of visual hallucinations, and to assess whether these techniques involve a shared underlying hallucinatory mechanism despite their differences. For each technique, we measured the frequency and complexity of the hallucinations produced, utilising button presses, retrospective drawing, interviews, and questionnaires. For both experimental techniques, simple hallucinations were more common than complex hallucinations. Crucially, we found that Ganzflicker was more effective than Ganzfeld at eliciting simple hallucinations, while complex hallucinations remained equivalent across the two conditions. As a result, the likelihood that an experienced hallucination was complex was higher during Ganzfeld. Despite these differences, we found a correlation between the frequency and total time spent hallucinating in Ganzflicker and Ganzfeld conditions, suggesting some shared mechanisms between the two methodologies. We attribute the tendency to experience frequent simple hallucinations in both conditions to a shared low-level core hallucinatory mechanism, such as excitability of visual cortex, potentially amplified in Ganzflicker compared to Ganzfeld due to heightened bottom-up input. The tendency to experience complex hallucinations, in contrast, may be related to top-down processes less affected by visual stimulation.

## Introduction

Hallucinations are commonly defined as perception in the absence of external stimuli^1,2^. In the case of visual hallucinations, these false percepts can range from simple—such as geometric forms^3–5^, to more complex, such as objects, human figures, or landscapes^6,7^. As well as occurring in pathological states such as schizophrenia^8^, Parkinson’s disease^9^, epilepsy^10^, migraine^11^ and vision impairment^12^, visual hallucinations can also be induced in non-pathological states—through the use of hallucinogens and psychedelics^13,14^, and via experimental manipulations such as states of sensory^15^ and perceptual^16^ deprivation (known as a ‘Ganzfeld’), and the use of flicker over closed^17,18^, or open^19,20^ eyes (also referred to as ‘Ganzflicker’ when viewed with open eyes). While experimentally-induced hallucinations are often referred to as ‘pseudo-hallucinations’ due to the participants’ awareness that the hallucinatory experience is not real, the percepts experienced can be very vivid and vision-like^19,20^. Experimental methods use different means of visual stimulation to induce hallucinations, and it is presently unclear to what extent these different approaches rely on different mechanisms and lead to differences in the nature and complexity of the resulting hallucinations^19,20^.

To interrogate this, we compared two experimental methods that differ vastly in the degree of bottom-up stimulation they involve: a salient, eyes-open visual flicker at a frequency appropriate for inducing visual hallucination (Ganzflicker), and visual and auditory perceptual deprivation (Ganzfeld). We tested whether increased bottom-up stimulation would alter the frequency and complexity of the hallucinations induced, and also whether the underlying mechanisms of the resulting hallucinations are shared across these methods despite the variations in visual stimulation.

Visual hallucinations can be divided into those of a ‘simple’ or ‘complex’ nature. Simple hallucinations contain abstract content such as colour alterations, elementary shapes, or geometric patterns. During a systematic study of the subjective effects of mescaline^21^, Heinrich Klüver outlined four key examples of simple hallucinations including tunnels, spirals, honeycombs and cobwebs. These form constants are cross-cultural and present across a multitude of hallucinatory states such as during psychedelic visual imagery^22^, migraine^11^, hypnagogia^23^ and flicker^17^, suggesting there may be a shared hallucinatory mechanism of relatively low-level origin underlying their occurrence. Indeed, neural network simulations suggest that form constants could arise from increased excitation in early visual cortices. When aberrant waves of excitation spread across early visual cortices, resulting in stripes on visual cortex, the transformation of these stripes from cortical space to retinal would result in characteristic geometric patterns resembling the aforementioned form constants^3–5^.

Complex hallucinations are those containing figurative elements such as faces, objects, or scenes. The representation of complex hallucinations has been associated with higher-order visual regions^7,24,25^. However, in psychedelic visual hallucinations, complex percepts resembling figurative constructs often also incorporate geometric elements, suggesting that there may not be a binary distinction between simple and complex hallucinations, and that lower-level visual areas may be concurrently active with higher-level visual areas during complex imagery^14^. This could suggest that simple hallucinations arise when activity is strongest in lower-level areas, whereas complex hallucinations arise when this activity travels up the visual hierarchy, either alone or in conjunction with top-down interpretative influences^18,26,27^. Another explanation is that complex hallucinations are more akin to mental imagery than to veridical vision^20^—i.e., they involve a top-down, or retro-hierarchical process^27–29^.

Our aim was to better understand both the nature of simple and complex hallucinations, and their underlying mechanisms. There are challenges in understanding the nature of these experiences, however, due to the difficulty in both eliciting and measuring hallucinations in a controlled laboratory environment with objective and methodologically sound techniques^30^. For instance, hallucinations experienced in pathological states can be uncommon and unpredictable—especially in clinical or laboratory settings. This is partly demonstrated by the number of single-subject case studies within the field, suggesting the difficulty of recruiting large numbers of patients experiencing visual hallucinations which can be measured on demand^31^. Similarly, psychedelics elicit a multitude of changes in conscious experience^32^ that make it difficult to draw conclusions about their specific effects on the visual system and the hallucinatory state. Greater control over the induction of hallucinations can instead be achieved in a laboratory environment using visual (and auditory) stimulation. Two methods of interest which are known to induce both simple and complex pseudo-hallucinations are high-frequency, eyes-open visual flicker (Ganzflicker) and perceptual deprivation (known as the ‘Ganzfeld’ effect). These methods have different stimulation conditions and have been suggested to rely on different underlying mechanisms^17,33–35^, based on which different predictions can be made surrounding the different types of hallucinations that may be elicited, and their complexity.

The term ‘Ganzfeld’ refers to a state of sensory homogeneity or perceptual deprivation (as opposed to sensory deprivation where stimulation is removed, e.g., via blindfold). The technique was first popularised by Metzger in 1930 (ganz = whole; feld = area, field)^36^, and traditionally refers to the exposure of an individual to homogenous, unstructured, sensory input (ganz = whole; feld = area, field), which can result in an altered state of consciousness^16,35^. In recent years, a ‘multimodal Ganzfeld’ has been induced by using halves of ping pong balls securely fastened over open eyes accompanied by light to achieve sensory homogeny^34,35,37–39^, combined with unstructured auditory stimulation such as white, brown, violet or pink noise to achieve auditory homogenisation^35,39^. After prolonged exposure to the Ganzfeld, both simple and complex pseudo-hallucinatory percepts have been reported to arise^34,35^. An fMRI study found reduced connectivity between the thalamus and primary visual cortex (V1) during a Ganzfeld compared to rest, which was attributed to a reduction in bottom-up signalling. When coupled with intact top-down fluctuations in activation, such a reduction in structured bottom-up signalling could cause sensory noise in early visual areas to be mistaken for signal, leading to hallucinatory experience^35^. Such a process may be especially heightened in the Ganzfeld, as internally generated percepts do not have to compete with veridical percepts^27^.

Flicker was first reported to induce hallucinations by Purkinje in 1819. While waving his hand between his eyes and sunlight, he reported ‘beautiful regular figures that are initially difficult to define but slowly become clearer’^7,40^. In more recent years, empty-field high-frequency flicker has consistently been found to produce hallucinations of both a simple^30,41–43^ and complex^19,20,44–46^ nature. Flicker stimulation can be implemented by viewing a flickering stimulus, i.e. viewing a flickering monitor with open eyes (‘Ganzflicker’^19,20^), or with flicker applied to closed eyes using a more powerful stroboscopic lamp, i.e. flicker light stimulation (FLS^17,45^). Both types of flicker-induced simple hallucinations have been proposed to utilise a rhythmic excitatory bias in early visual cortices^43,47^. In this process, wave patterns of excitation, akin to those associated with geometric Klüver-like form constants, are thought to encourage neural entrainment; the synchronisation of the brain’s endogenous neural oscillations (e.g., the alpha rhythm) to an endogenous external stimulus such as the flicker^17,18,33,48^. In line with this, several studies have shown that simple hallucinations are most frequent when flicker is presented at the alpha-wave frequency of approximately 10 Hz^17,49^ (although hallucinations also occur at other frequencies^13,14^). In the case of flicker-induced complex hallucinations, it has been suggested that basic hallucinatory forms could act as building blocks for interpretive top-down influences^18^.

### Aims and hypotheses

Given the above differences in both the pattern of visual stimulation and the proposed mechanisms of flicker- and Ganzfeld-induced hallucinations, we sought to directly compare the nature and frequency of the visual pseudo-hallucinations produced by these methods. We did this by comparing eyes-open viewing of a flickering monitor (Ganzflicker) and eyes-open-but-covered perceptual deprivation (Ganzfeld).

We present three hypotheses regarding the interplay of bottom-up and top-down dynamics in generating hallucinations of differing complexities, during the Ganzflicker and Ganzfeld techniques:

- **H1**: Simple hallucinations are primarily driven by bottom-up inputs.
- **H2**: Complex hallucinations are primarily driven by top-down mechanisms.
- **H3**: Ganzflicker and Ganzfeld share a common underlying mechanism.

These hypotheses give rise to the following predictions:

- Prediction 1: if H1 is true then the increased bottom-up input from Ganzflicker (i.e., viewing a flickering monitor) should give rise to more simple hallucinations than the Ganzfeld. Simple hallucinations during Ganzfeld, a technique involving perceptual deprivation, may rely more on inherent visual cortex excitability, which would likely be more variable between individuals. The alternative, if H1 is not true, is that the number of simple hallucinations across Ganzflicker and Ganzfeld should be similar.
- Prediction 2: if H2 is true then we would expect the frequency of complex hallucinations to be unrelated to the frequency of simple hallucinations. This may manifest in multiple ways. For instance, there may be more complex hallucinations during Ganzfeld due to a lack of bottom-up input competing with top-down drive, or the frequency of complex hallucinations across Ganzflicker and Ganzfeld may be similar, unaltered by visual stimulation conditions. The alternative, if H2 is not true, is that the greater bottom-up stimulation of the Ganzflicker should lead to an equivalent increase in both simple and complex hallucinations, relative to the Ganzfeld.
- Prediction 3a: if H3 is true in a strong form, and the two conditions involve identical mechanisms, then there should be a similar pattern of simple versus complex hallucinations in the two conditions (please note that this would also mean that Predictions 1 and 2 are not met), and a high correlation between the frequency of hallucinations across the two methods.
- Prediction 3b: Even if prediction 3a is not met, it could still stand that a more moderate version of H3 is true. That is, the two conditions may share an underlying sub-process rather than fully shared mechanisms. In this case, we might expect distinct patterns of simple vs. complex hallucination rates, but measures of hallucinatory experience should still correlate between the Ganzfeld and Ganzflicker i.e., people who are more hallucination-prone and experience more hallucinations from Ganzflicker should also experience more hallucinations during the Ganzfeld.

## Materials and methods

### Sample

Thirty participants (21 female, nine male) with a mean age of 29.5 ± SD 8.03 years (range 18–50 years) completed the study. This sample size was intended to match or exceed the samples of previous studies on experimentally-induced hallucinations^17,18,38,42,44^. None of the participants had a history of neurological disorder, including any history of seizure or adverse experience to flashing or flickering lights. Participants were recruited through university recruitment systems and word of mouth. All participants provided informed consent. The experimental procedure was approved by the Experimental Psychology Ethics Committee at the Division of Psychology and Language Sciences, University College London. The research was carried out in accordance with the tenets of the Declaration of Helsinki.

### Study design

The study had a counterbalanced repeated measures design with all participants undergoing 15 min of Ganzflicker and 25 min of Ganzfeld. The duration of the Ganzfeld was determined in line with previous literature^35,38^, and a pilot study which suggested that it often took participants longer to see pseudo-hallucinatory percepts in the Ganzfeld than during Ganzflicker. The duration of Ganzflicker was chosen based on previous literature^18,19^ and a pilot study in which some people considered continuous extended flicker above this duration uncomfortable. In both conditions, participants were provided with a keyboard and asked to indicate both the onset and offset of any perceived visual pseudo-hallucination (outlined in *Response measures—Button press and* drawing*)* and to provide a brief prompt of what they saw, which the experimenter noted.

**Figure 1:**
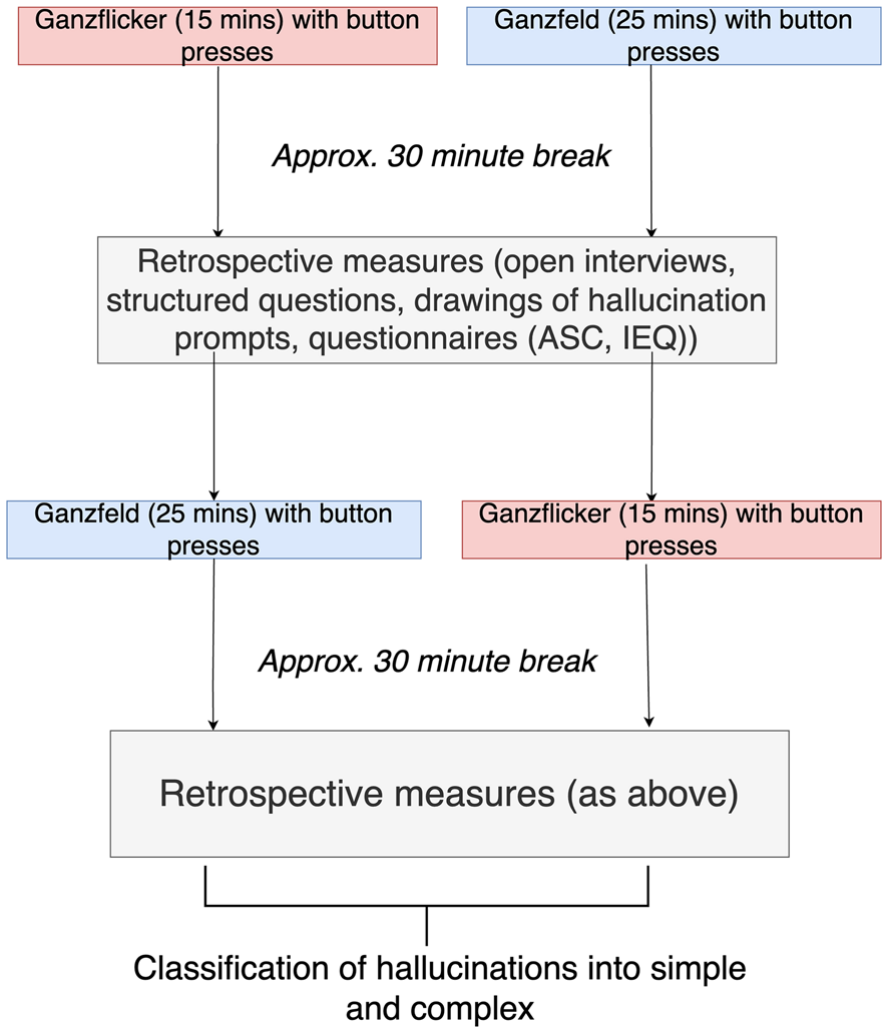
Study design. 30 participants were split into two groups of 15. Utilising a counterbalanced repeated measures design, each group experienced both Ganzflicker and Ganzfeld experimental sessions; one group started with 15 min of Ganzflicker followed by 25 min of Ganzfeld, while the other group followed the reverse order. A button-press paradigm was utilised within each experimental condition. Using a keyboard, participants signalled the onset and offset of their hallucinations. Post-hallucination offset, participants provided brief verbal prompts detailing the content of their hallucinatory experiences. Approximately a thirty-minute break separated each experimental condition. During this interval, participants engaged in an open interview and responded to structured questions surrounding their level of sleepiness and the perceived impact of button presses on their hallucinatory experiences. Additionally, participants drew their previous hallucinatory prompts and completed two retrospective questionnaires, the ASC and the IEQ. After the experimental session had ended, experimenters later used these prompts and drawings to classify hallucinations into simple and complex. ASC—Altered States of Consciousness (Rating Scale)**;** IEQ—Imagery Experience Questionnaire.

There was an approximately thirty-minute break between conditions to allow for a wash-out period. During this time, participants a) drew their hallucinatory experiences with respect to their given prompts, b) rated their sleepiness and their perception of how much they felt the button press interfered with their experience, c) underwent an open interview, and d) completed two retrospective questionnaires (covered in detail in Supplementary Materials—*Questionnaires*, Supplementary Table 1 and Supplementary Table 2). The study design is given in Fig. 1.

### Experimental procedures

#### Instructions

Prior to the experiment, participants were given brief examples of the types of visual experiences they might experience in order to facilitate homogenous reporting of hallucinations. Participants were informed that they could see any combination of a) simple or abstract hallucinations such as colours, movement, patterns such as kaleidoscopes, or shapes or b) complex or figurative hallucinations, such as objects, animals, faces or scenes. They were also told that they may not see anything at all. They were informed that these visual experiences were of a ‘pseudo-hallucinatory’ nature, and that they would be aware that what they were seeing was not real. They were encouraged to be patient while waiting for any experiences to arise, especially with the Ganzfeld condition. These instructions were provided to ensure that participants reported all visual phenomena experienced, even those that may have been more minor than they were expecting, but also to allow them to keep an open mind during the experiment.

Participants were asked not to engage in any active daydreaming or mental imagery, and to passively watch any observations, making sure to pay attention to them and allow them to evolve if necessary. To this end, participants were asked not to verbalise their hallucinations until the experience faded away, however, they were not interrupted if they began verbalising their experience while the hallucination was ongoing. Participants were instructed to keep their eyes open for both conditions. Further experiment details are given in the Supplementary Materials—*Further experiment details.*

#### Ganzflicker

Participants were seated in a darkened and soundproofed room, at 70 cm from a 32″ LED backlit LCD monitor (60 Hz frame rate; Cambridge Research Systems BOLDscreen). The screen flickered an alternating black and red display (as in previous literature^19,20^) for 15 min. The screen flickered at a frequency of 10 Hz, which was chosen based on multiple studies suggesting that flicker between 8 and 12 Hz, and in particular 10 Hz, is a suitable frequency for eliciting visual pseudo-hallucinations^45,46,49^. The luminance of the red display was 69.93 cd/m^2^, and the luminance of the black display was 0.38 cd/m^2^. Stimuli were coded using Psychtoolbox-3 running on MATLAB 2021b. Participants were trained on using the button press by first undergoing a 30 s practise run during which they saw the flickering stimuli and practised pressing the appropriate buttons and verbalising their experience. This was normally sufficient time for a simple hallucination to occur, and therefore to facilitate comprehension of the task. If participants were unclear on the instructions, they were allowed another practice run until they understood the task at hand. Auditory brown noise was played through noise-cancelling headphones and adjusted per participant to a volume which was comfortable, but loud enough to block out any external sound^37^.

#### Ganzfeld

Orange-coloured ping-pong balls were halved and taped securely over the eyes using medical tape. The visual field was illuminated by a warm white light (Lumary 24W Smart LED Flood Light). Participants were seated approximately 30 cm from the light. Auditory brown noise was played through noise-cancelling headphones using the protocol outlined above. While seated, but without their eyes covered, participants practised using the keyboard to report possible hallucinatory experiences.

### Response measures

#### Button press and drawing

For both Ganzflicker and Ganzfeld conditions, participants were asked to press the left arrow button on a keyboard when they felt a hallucinatory experience appearing, and to indicate via the right arrow button when the experience had faded away. These button presses provided an indication of the number and the duration of hallucinations experienced by participants. Once the right arrow button was pressed (indicating the percept had completely faded), participants were instructed to verbalise in as few words as possible what they saw. This prompt was noted by the experimenter. Participants were told that these notes would be used as a prompt for their drawings later. From the button-press data, we counted the number of hallucinations, which were transformed into a rate of hallucinations per minute (i.e. the number of hallucinations divided by 15 for the Ganzflicker condition and 25 for the Ganzfeld condition, given their respective durations). We also acquired the average duration of hallucinations (i.e. the time from the onset button press to the offset button press), and the total proportional time spent hallucinating (the total summed duration of hallucinations, divided by the length of the condition).

##### Classification of hallucinations

After the experiment, participants were given prompts to draw impressions of their hallucinatory experiences—either using paper and coloured pens in earlier iterations of the experiment, or via an iPad (9th generation) in later experiments. These drawings, combined with the associated prompts, gave the experimenter an idea of the nature of the hallucinations experienced and informed their classification as ‘simple’ or ‘complex’.

Simple hallucinations were defined as any descriptions and corresponding drawings of colours, shapes, or patterns including characteristic Klüver constants (for instance ‘blob’, ‘blue’, ‘tunnel’, ‘grid’). Complex hallucinations were defined as those with corresponding semantic value (for instance ‘dog’, ‘flower’, ‘galaxy’, ‘face’). When participants felt unable to draw their given prompts (i.e. ‘unsure’, ‘moving’, ‘pulsating’, ‘don’t know’), they were classified as simple hallucinations. We made this classification with the assumption that complex hallucinations would involve rich and detailed content that could be readily described, whereas simple hallucinations involve more basic or abstract visual phenomena that can be challenging to put into words due to their abstract or non-specific nature. While we acknowledge that prompts like 'unsure' or 'don't know' indicate uncertainty, we opted to retain these responses as 'simple' hallucinations to capture the full range of experiences reported by participants, even when they struggled to provide a detailed description. In this sense, we were conservative in our classification of complex hallucinations in particular—concordance between the prompt and the drawing was required to be appropriately classified as a complex hallucination. Thirty such examples of hallucinations and their classifications are given in Supplementary Table 3. To further compare phenomenology across visual stimulation conditions, we also used the words provided for these prompts for a word frequency analysis.

#### Questionnaires

Two abridged questionnaires were used to retrospectively assess the subjective experience of participants. We used the questionnaire measures as validation for our button press data.

We used the Altered States of Consciousness Rating Scale (ASC-R^50^), a well-validated 94 item self-report scale for the retrospective assessment of pharmacological and non-pharmacological induced altered states of consciousness. We chose questions primarily from the Elementary Imagery and Complex Imagery dimensions in line with our research question to interrogate the nature of participants’ subjective experience. Further rationale for the items chosen are described in the Supplementary Materials—Methods, *Questionnaires*, and specific items are shown in Supplementary Table 1.

Though the ASC-R measures visual phenomenology, many of the items are non-specific to hallucination research. Therefore we also utilised questions from the newly developed Imagery Experience Questionnaire (IEQ^51^). The IEQ was designed to capture the subjective experience of visual psychedelic experiences more accurately and with greater depth, and many of the items within it are relevant for visual experiences induced by non-pharmacological altered states of consciousness, such as Ganzflicker and perceptual deprivation. The dimensions from the IEQ are divided into Complexity, Content and Progression. We only used the Complexity and Progression items. In line with our research question, we carried out analyses pertaining to hallucination complexity by separating items from the Complexity dimension of the IEQ into simple (items 1, 2, 3 and 4) and Complex (items 5, 6, 7 and 8). The relevant items used within the study are in Supplementary Table 2.

#### Rating scales and open interview

Immediately after the experiment, participants were asked to score their a) sleepiness and b) opinion on how much they felt the button press and talking about their experience during the experiment interfered with their visual hallucinations on a 7-point Likert scale from 0 (Not at all) to 6 (very much so).

Participants also underwent an unstructured, open interview, where they were asked ‘Please tell me anything you feel might be relevant to your Ganzflicker or Ganzfeld experience. This could include the visual elements of your experience, but also how you felt. If you feel it is relevant, you can also talk about how tired you were during the experience and how much you feel the button press and discussing your hallucinations during the experiment impacted your experience’. We conducted word frequency analyses and exploratory word clouds using the words collected per visual stimulation condition.

### Statistical analyses

Shapiro–Wilk normality tests were used to assess the normality of our dependent variables. Of our dependent variables, only ASC and IEQ scores were normally distributed. To assess whether simple and complex hallucinations were more likely to occur in Ganzflicker compared to Ganzfeld in our frequency data (hallucinations per minute), we used negative binomial distribution mixed effects models with condition (Ganzflicker vs Ganzfeld) and complexity (simple vs complex) as fixed-effect predictors alongside an interaction term. We allowed intercepts to vary randomly per participant to accommodate individual heterogeneity, and included an offset term to account for the duration of each respective experiment. We modelled the hallucination frequency data with a negative binomial distribution as they were count data (transformed to frequencies) composed of only non-negative integers with a positive skew and many zeros^52^.

We used contrasts to test for differences between simple and complex hallucinations within and across conditions (i.e., main effects). We separately checked for interaction effects in order to test whether simple versus complex hallucinations are more likely to occur in one condition over another. We report the beta parameters (or the exponential of the beta parameters) corresponding to these effects and their significance. To supplement these analyses, we also carried out a Bayesian negative binomial analysis of our count data using the *brms*^53^ package in R. Bayes factors (BF) were then calculated using the Savage-Dickey method^54^. We further carried out a Chi-squared test to examine differences in number of hallucinations based on both experimental condition and hallucination complexity.

We used a similar approach to test for differences in average duration of hallucinations across visual stimulation conditions and for different complexities, and to test for differences in questionnaire scores across these factors. For duration data we carried out gamma mixed effects models with a log link because this captures the distribution of the data used (non-negative, positively skewed), again taking condition and complexity as fixed-effects predictors, and participant number as a random-effects predictor with varying intercepts. As our questionnaire data (ASC and IEQ) were normally distributed, we used a linear mixed effects model, again with the same model structure.

When any other statistical methods were used, these are noted in the “Results” section. In brief, for comparisons between two groups, t-tests (or Wilcoxon signed-rank tests in the case of non-parametric data) were used. When testing for correlations in hallucination measures across Ganzfeld and Ganzflicker we used Spearman’s rank correlations for non-parametric data, and Pearson’s correlation for parametric data.

All analyses were carried out in R version 4.3.

#### Exploratory qualitative analyses

##### Word frequency analysis

We used a word frequency analysis to identify the most frequent words used as interview prompts by participants during the hallucinatory conditions. We tokenised all of the prompts and removed stop words that were considered to add no significant meaning to the analysis (i.e. ‘and’, ‘to’) using the *stop_words* dataset from the R package *tidytext*^55^. We formulated stop-words specific to the study ("screen", "orange", "black", “red”, “Ganzfeld”, “hallucination”, “ping pong balls”, “flashing”, “flicker”) and combined these with the stop_words dataset. For each remaining word after the removal of stop words, we counted the frequency and the percentage of total word mentions in each respective condition.

##### Word clouds

Open interview data was used to create word clouds using the R package *wordclouds* after the removal of the previously mentioned stop words. Prior to this, the data was stemmed so words that may have appeared as plurals would only appear in their singular form (i.e. shapes = shape; colours = colour; patterns = pattern).

## Results

### Individual examples

**Figure 2:**
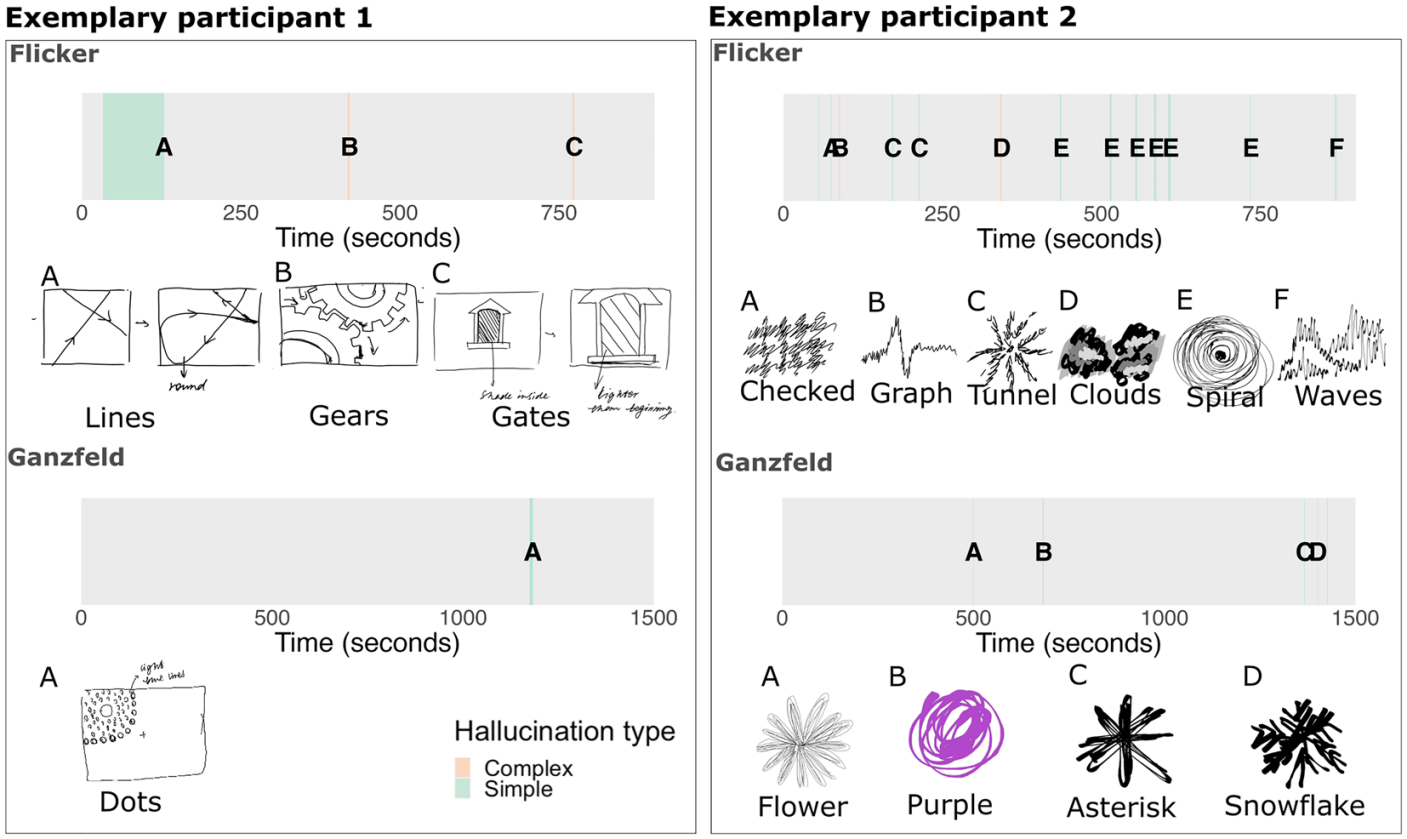
Individual time series for Ganzflicker and Ganzfeld for two exemplary participants. Grey bars depict the time course of each trial, with coloured regions showing the onset and duration of simple (green) and complex (orange) hallucinations elicited in each condition. Hallucinations in each condition are marked with letters, with the corresponding drawings that were subsequently produced by participants shown below the time series accompanied by the abbreviated prompts provided.

The plots in Fig. 2 show examples of the time series and accompanying drawings and prompts for the hallucinations that two participants experienced in the Ganzflicker and Ganzfeld condition. These two participants demonstrate the variability and the spectrum of hallucinatory experience that participants could encounter, ranging from very minor perceptual phenomena to multiple complex hallucinations.

### Primary analyses

**Figure 3:**
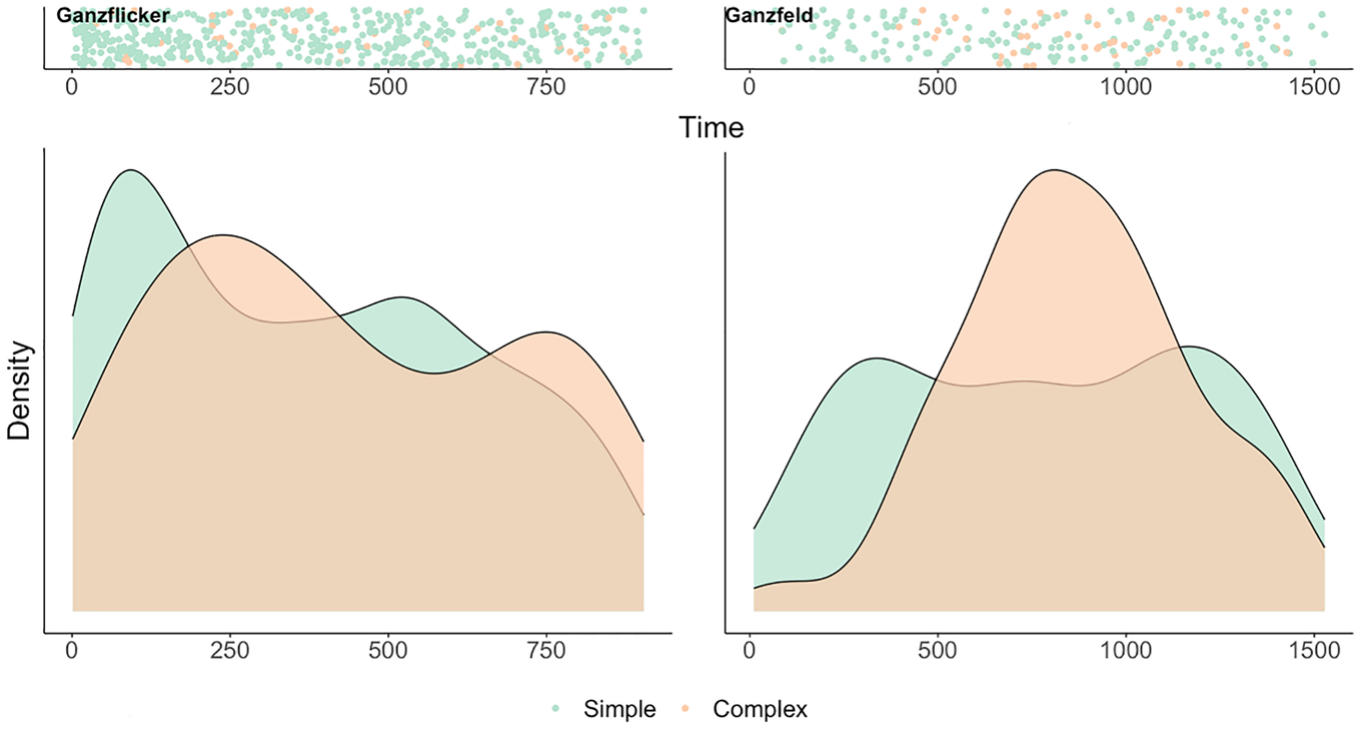
The time course of simple (green) and complex (orange) hallucinations elicited through Ganzflicker (left) and Ganzfeld (right) pooled across participants. Scatter plots (shown in upper panels) illustrate the onset (time of first button press provided by participant) of simple and complex hallucinations across the time course of Ganzflicker (left, 900 s) and Ganzfeld (right, 1500 s). Lower panels show the corresponding normalised probability distributions (the probability of a hallucination occurring with each distribution peaking at 1) in Ganzflicker (left) and Ganzfeld (right). All plots are *N* = 30.

To provide a generalised overview of the time course of simple and complex hallucinations in Ganzflicker and Ganzfeld conditions, we begin with an exploratory analysis of the probability distributions of simple and complex hallucinations (as defined as the time of a button press onset) in Fig. 3. During Ganzflicker (A), simple hallucinations tended to have an earlier onset (peaking at 94 s) than complex hallucinations (peaking at 266 s). This pattern was also present for the Ganzfeld (B), though overall onset times were later than during the Ganzflicker condition, with simple hallucinations peaking first at 269 s and complex hallucinations peaking at 828 s. Interestingly, simple hallucinations show a bimodal distribution in both the Ganzflicker and the Ganzfeld, with a secondary peak at 520 s in the Ganzflicker and a secondary peak at 829 s in the Ganzfeld. Complex hallucinations also showed a secondary peak during Ganzflicker at 749 s.

We next consider the quantitative measures relevant to our hypotheses. A summary of the descriptive statistics (mean and standard deviation) for these measures can also be found in Supplementary Table 4.

**Figure 4:**
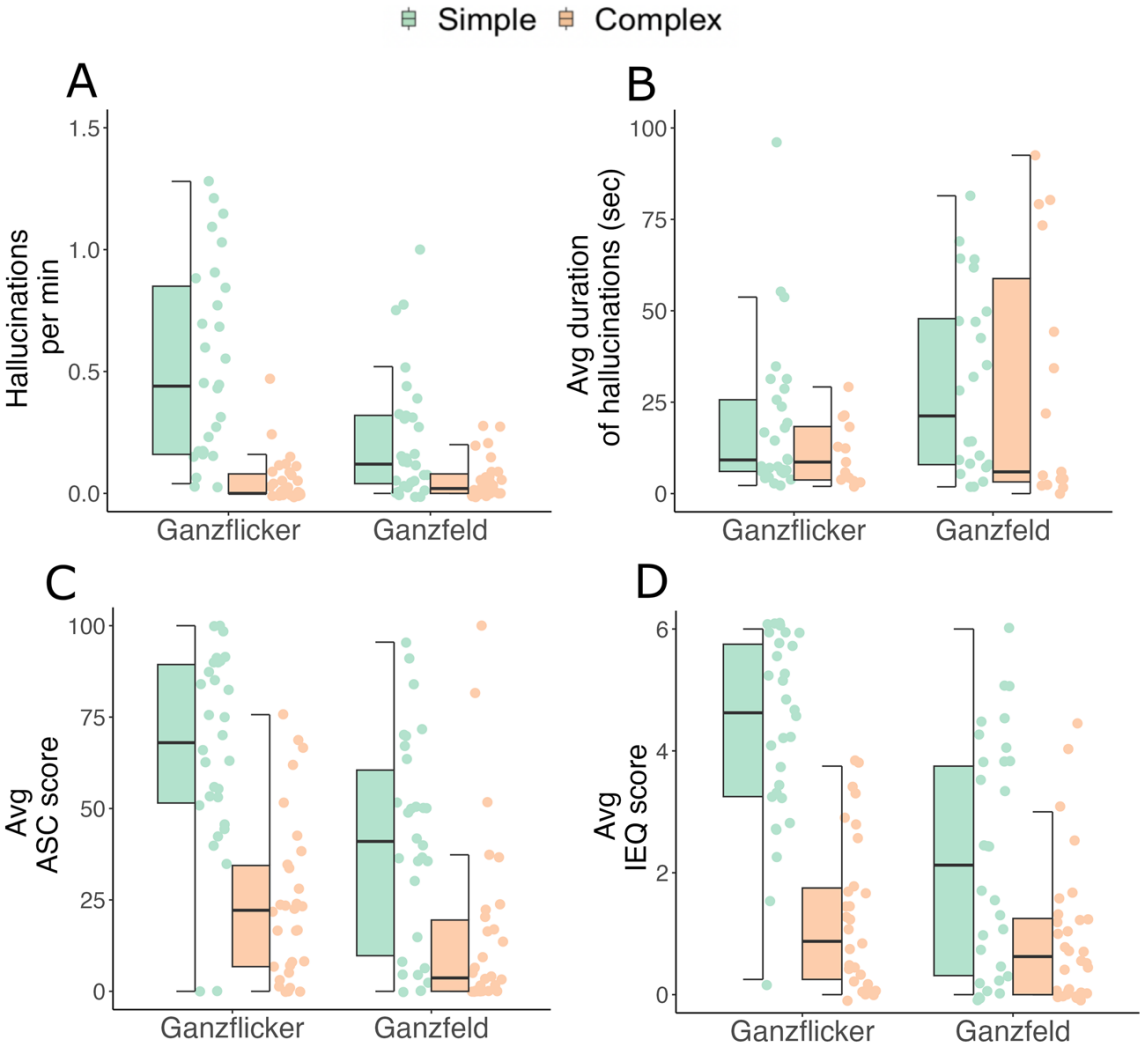
(**A**) The frequency of simple (green) and complex (orange) hallucinations, plotted as the total number of hallucinations divided by the duration of experimental conditions (Ganzflicker—15 min.; Ganzfeld – 25 min.) on the y-axis, across experimental conditions (x-axis) (**B**) The average duration of hallucinatory periods in seconds (y-axis) of simple and complex hallucinations across conditions (x-axis) (**C**) Average ASC scores (y-axis) split between Elementary Imagery (green) and Complex Imagery dimensions (orange) across experimental conditions (x-axis) (**D**) Average IEQ scores (y-axis) split between Simple Imagery (green) and Complex Imagery components (orange) across experimental conditions (x-axis). All plots are *N* = 30. ASC—Altered States of Consciousness (Rating Scale)**;** Avg—average; IEQ—Imagery Experience Questionnaire; min—minute. The y-axis of plot A has been capped at 1.5 for visualisation purposes; two data points exceeded this value. Similarly, the y-axis of plot B has been capped at 100 for visualisation purposes, two data points exceeded this value.

We first compared the frequency of simple and complex hallucinations across Ganzflicker and Ganzfeld using negative binomial models (Fig. 4A). Within conditions, the frequency of simple hallucinations was greater than the frequency of complex hallucinations for both Ganzflicker (Incidence rate ratio (IRR) = 13.61, SE = 3.73, Z = 9.54, *p* < 0.001) and Ganzfeld (IRR = 3.87, SE = 1.35, Z = 3.27, *p* < 0.001).

Across conditions, the frequency of simple hallucinations was higher in Ganzflicker compared to Ganzfeld, providing support for H1 (IRR = 5.75, SE = 1.29, Z = 7.80, *p* < 0.001). In contrast, the frequency of complex hallucinations did not differ in Ganzflicker compared to Ganzfeld (IRR = 0.61, SE = 0.19, Z = −1.58, *p* = 0.115), and there was also a significant interaction between condition and complexity (IRR = 0.28, SE = 0.11, Z = −3.27, *p* = 0.001), supporting H2. We supplemented these analyses with a Bayesian negative binomial analysis, which also provided support for our key hypotheses (Supplementary Table 5).

Descriptively, the ratio of simple to complex hallucinations was 11.5:1 during Ganzflicker, whereas it was 4:1 during Ganzfeld. To assess the difference between these ratios, we used a Chi-squared test which showed a significant association between hallucination complexity and experimental condition (X2 (1, 735) = 20.82, *p* < 0.001). The difference in these ratios is primarily driven by the increased rate of simple hallucinations in the Ganzflicker, while complex hallucinations remain largely unchanged, again providing evidence for H2.

To summarise, the frequency of experiencing a simple hallucination was greater than that of experiencing a complex hallucination during both Ganzflicker and Ganzfeld. However, only simple hallucinations occurred more frequently in Ganzflicker than in Ganzfeld. Within a given condition, the consequence is that the likelihood of experiencing a complex hallucination was significantly higher during Ganzfeld than in Ganzflicker.

We then tested whether the average duration of hallucinations (i.e., the time from start to end button press) varied by content and visual stimulation condition using gamma mixed effects models with a log link function (Fig. 4B). We did not have any a priori predictions about the results of this analysis. Across conditions, hallucinations were shorter in Ganzflicker compared to Ganzfeld for both simple (exp(β) = 0.45, SE = 0.03, T = −11.48, *p* < 0.001) and complex (exp(β) = 0.64, SE = 0.11, T = -2.57, *p* = 0.031) hallucinations. Within conditions, there was no significant difference in the duration of simple versus complex hallucinations during Ganzflicker (exp(β) = 0.95, SE = 0.13, T = −0.35, *p* = 0.730). Simple hallucinations were slightly but significantly longer than complex in the Ganzfeld condition (exp(β) = 1.42, SE = 0.20, T = 2.54, *p* = 0.011). There was also a significant interaction between condition and complexity (exp(β) = 0.67, SE = 0.13, T = −2.09, *p* = 0.037). Thus, the average duration of a reported hallucination was longer during Ganzfeld than in Ganzflicker, and simple hallucinations tended to be longer than complex hallucinations, particularly in the Ganzfeld.

**Figure 5:**
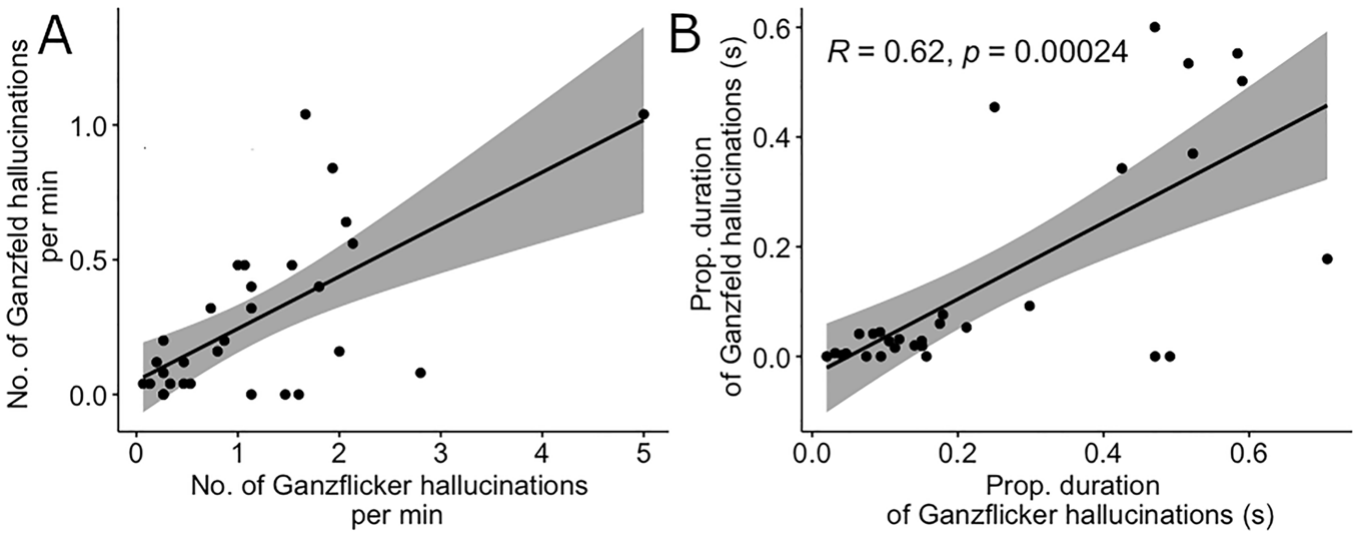
Scatterplots showing (**A**) the relationship between the number of Ganzflicker hallucinations per minute (x-axis) and the number of Ganzfeld hallucinations per minute (y-axis); (**B**) The total proportional time spent hallucinating (total time spent hallucinating divided by the duration of the experimental condition) in Ganzflicker (x-axis) compared to Ganzfeld (y-axis) with associated trend line (black) and Spearman’s rank correlation testing (95% CI grey shading) for *N* = 30. Prop—proportional.

To investigate whether people who reported more hallucinations when looking at Ganzflicker also reported more hallucinations during the Ganzfeld (H3), we ran Spearman’s rank correlation analyses between our button press measures. The correlation between the frequency of Ganzflicker hallucinations and the frequency of Ganzfeld hallucinations was significant (r_s_(28) = 0.55, *p* = 0.0016, Fig. 5A), consistent with a common mechanism between the two techniques. When combining the frequency and duration of hallucinations into a combined measure of overall time spent hallucinating during each condition (total proportional duration time spent hallucinating), we further found a positive correlation between experiences during the Ganzflicker and Ganzfeld (r_s_(28) = 0.62, *p* < 0.001, Fig. 5B). The correlation between the average duration of Ganzfeld and Ganzflicker hallucinations was not statistically significant (r_s_(28) = 0.35, *p* = 0.056).

#### Questionnaire validation

Button presses provide a direct and quantifiable measure of hallucination frequency, onset, and offset. However, they may be affected by non-experiential variables such as the criterion for when to press (i.e., some people may press for a very faint experience, while others may press only for a very vivid experience). Therefore, we validated our button press findings with subjective hallucination intensity as measured through retrospective questionnaires, using the ASC and IEQ.

Using linear mixed effects models, we show that within conditions, Elementary Imagery scores (ASC) and Simple Imagery scores (IEQ) were higher than Complex Imagery scores in both Ganzflicker (ASC: β = 41.60, SE = 5.79, T = 7.18, *p* < 0.001; IEQ: β = 3.08, SE = 0.32, T = 9.70, *p* < 0.001) and Ganzfeld (ASC: β = 25.15, SE = 5.79, T = 4.34, *p* < 0.001; IEQ: β = 1.29, SE = 0.32, T = 4.06, *p* < 0.001), as plotted in Fig. 4C,D. Across conditions, Elementary Imagery (ASC) and Simple Imagery (IEQ) scores were greater in Ganzflicker compared to Ganzfeld (ASC: β = 25.62, SE = 5.79, T = 4.42, *p* < 0.001; IEQ: β = 2.11, SE = 0.32, T = 6.63, *p* < 0.001), providing support for H1. Conversely, Complex Imagery scores were not higher in Ganzflicker than in Ganzfeld (ASC: β = 9.17, SE = 5.79, T = 1.58, *p* = 0.116; IEQ: β = 0.32, SE = 0.32, T = 1.00, *p* = 0.321). There was also a significant interaction effect between condition and complexity (ASC: β = 16.45, SE = 8.2, T = 2.01, *p* = 0.047; IEQ: β = 1.79, SE = 0.45, T = 3.98, *p* = 0.001), providing support for H2. These results show highly consistent patterns of results not only across the two questionnaires, but also across questionnaires and button-press data, providing independent evidence for H1 and H2.

We also carried out correlations between total ASC and IEQ scores across conditions in order to validate our observed correlations between Ganzfeld and Ganzflicker button press measures (H3b). We found a positive correlation between average IEQ scores pertaining to Ganzflicker and average IEQ scores pertaining to Ganzfeld (r(28) = 0.55, *p* = 0.0015). The correlation in the ASC was not statistically significant (r(28) = 0.28, *p* = 0.14). Since the IEQ is not a validated measure, we tested its validity in the present study. In line with the corresponding results described above, we found a correlation (Pearson’s) with the ASC-R, a well-validated questionnaire for both Ganzflicker (Supplementary Fig. 1A; r(28) = 0.58, *p* < 0.001) and Ganzfeld (Supplementary Fig. 1B; r(28) = 0.78, *p* < 0.001). In addition, a visualisation of both questionnaires across both conditions is given in Supplementary Fig. 2.

Thus, retrospective questionnaire measures largely replicated the results obtained via button press and hallucination prompts. In order to explore whether our methodology played a role in producing these results, we also assessed whether participants felt that the button press interfered with their experience, e.g., through decreasing the duration of hallucinations. This may be a particular problem in the Ganzfeld, which may require a higher degree of immersion for hallucinations to occur^38^. We did not observe any evidence that the perceived perception of the interference of the button presses interfered with the occurrence of hallucinations, as there were no correlations (*p* > 0.05) between our button-press measures and participants’ perception of the interference of the button press, nor were there any differences between perception of button press interference experience across the conditions (Full details given for analysis in Supplementary Materials—*Button press interference and sleepiness*, V = 74, *p* = 0.77, Supplementary Fig. 3B).

#### Additional analyses

##### Word frequency analysis

**Figure 6:**
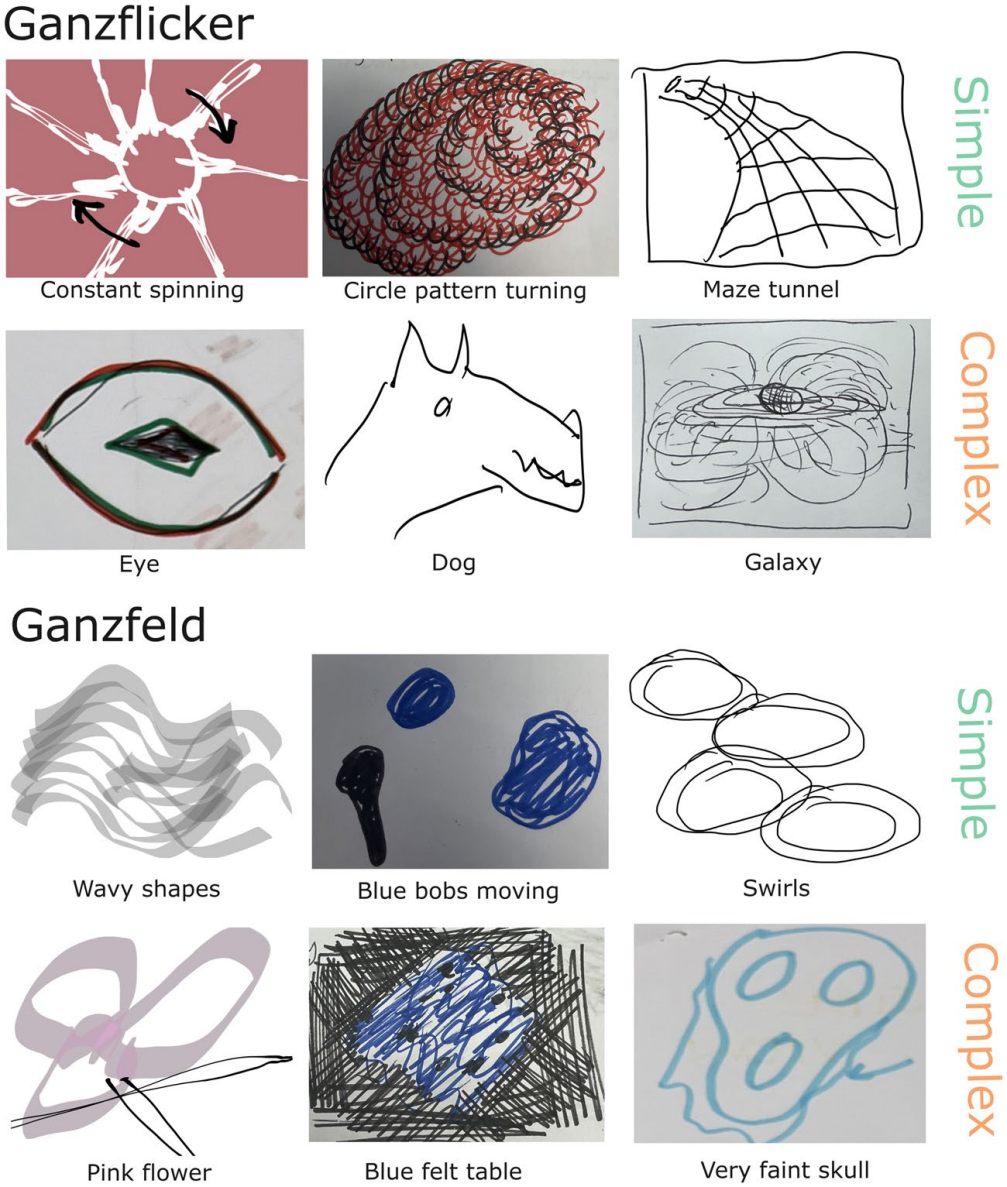
Examples of the common simple and complex hallucinations as given by prompts provided by participants during experimental conditions in Ganzflicker and Ganzfeld. Abbreviated prompts accompany drawings.

An exploratory word frequency analysis was undertaken to identify common words within the prompts given by participants during the button-press component of the experiment. The ten most frequently reported words for simple and complex hallucinations in the Ganzflicker and Ganzfeld condition are given in Table 1. Examples of the drawings of the most frequent words are given in Fig. 6. Interestingly, though there are words of a dynamic nature (i.e. spinning, moving, pulsating) in both conditions, these were more common in the Ganzflicker than the Ganzfeld. In addition, there are references to geometric form constants during Ganzflicker (i.e. tunnel, galaxy), whereas the percepts described during the Ganzfeld tend to lack this geometric regularity (i.e. swirls, bubbles, cloud). Despite the warm hued tint of the light stimulation during Ganzfeld, words associated with colour tended to be more cool toned (blue, green).

**Table 1:** Word frequency analysis illustrating the frequency of words used in the hallucinatory prompts given by participants during Ganzflicker (first two columns)) and Ganzfeld (last two columns)), split out by hallucination complexity (simple/complex).

Ganzflicker		Ganzfeld	
Simple, n (%)	Complex, n (%)	Simple, n (%)	Complex, n (%)
Line(s), 64 (4.62)	Dog, 6 (5.00)	Moving, 18 (3.22)	Flower, 4 (2.92)
Moving, 60 (4.33)	Eye(s), 5 (4.17)	Shapes, 16 (2.86)	Blue, 3 (2.19)
Circles, 35 (2.53)	Galaxy, 4 (3.33)	Lines, 11 (1.97)	Faint, 3 (2.19)
Spinning, 26 (1.88)	Shape, 4 (3.33)	White, 11 (1.97)	Green, 3 (2.19)
Middle, 24 (1.73)	Indistinct, 3 (2.50)	Colour, 10 (1.79)	Shapes, 3 (2.19)
Top, 24 (1.73)	Top, 3 (2.50)	Pulsating, 10 (1.79)	Car, 2 (1.46)
Tunnel, 24 (1.73)	Bridge, 2 (1.67)	Swirls, 9 (1.61)	Clouds, 2 (1.46)
White, 23 (1.66)	Butterfly, 2 (1.67)	Bubbles, 8 (1.43)	Colours, 2 (1.46)
Circle, 22 (1.59)	Half, 2 (1.67)	Blue, 7 (1.25)	Hollow, 2 (1.46)
Left, 21 (1.52)	Planet, 2 (1.67)	Circle, 7 (1.25)	Leaf, 2 (1.46)

##### Word clouds

**Figure 7:**
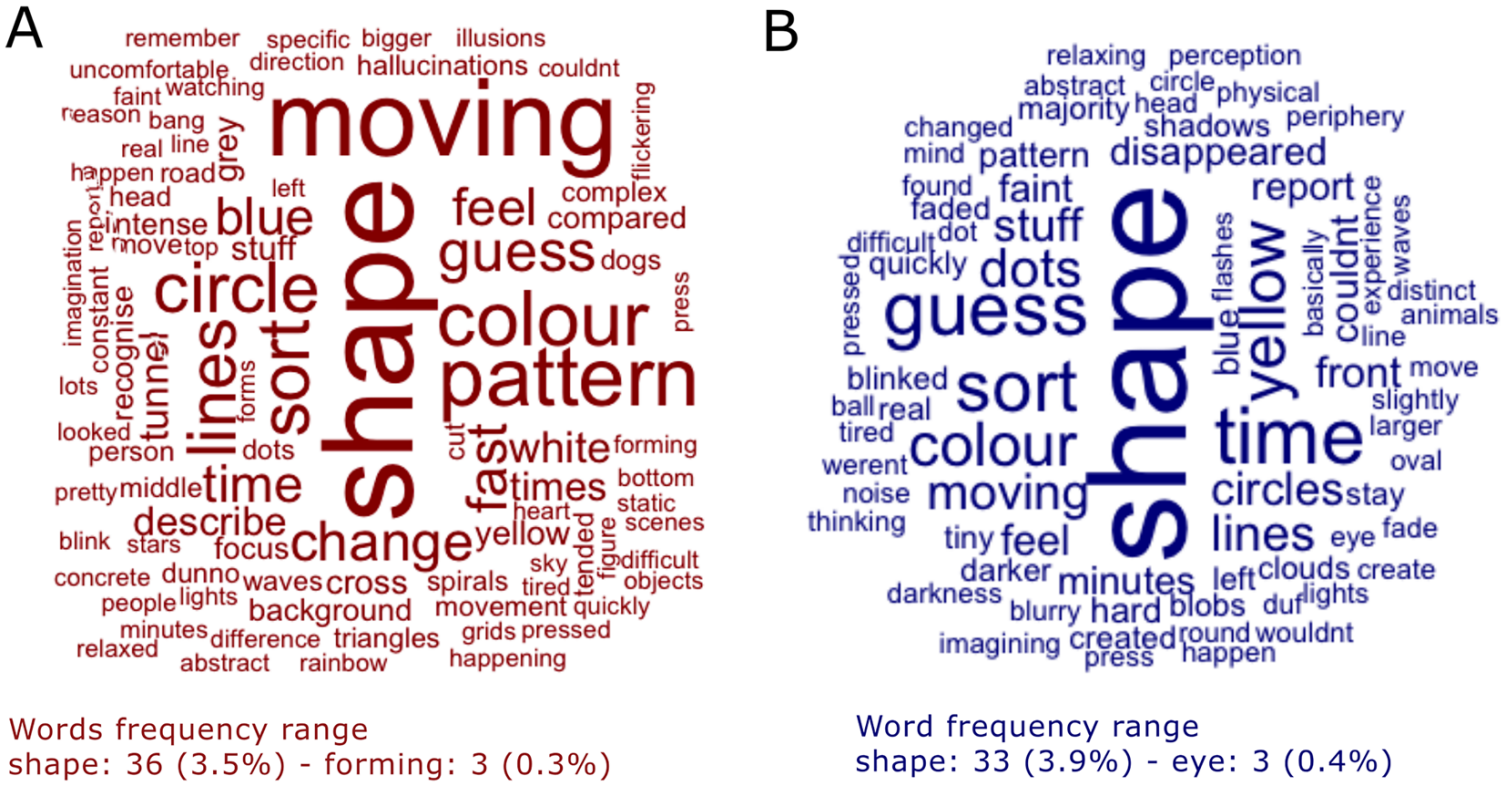
Word clouds for Ganzflicker (**A**, red) and Ganzfeld (**B**, blue) created from open interview data. Size of words is indicative of frequency of usage in open interview data.

Exploratory word clouds created from the open interview data are given in Fig. 7 for Ganzflicker (A) and Ganzfeld (B). A complementary word frequency table is given in Supplementary Table 6. The words utilised in these word clouds were those used three or more times overall (0.29% of the time in Ganzflicker and 0.36% of the time in Ganzfeld). The maximum frequency of words used was 3.46% (36 times) in Ganzflicker and 3.92% (33 times) in Ganzfeld.

Interestingly, references to form constants and geometric percepts are present in the Ganzflicker condition (tunnels, spirals, cross, grids), but not in the Ganzfeld. Minor perceptual phenomena which could be related to phosphenes (line, round, oval, dots, circle(s)) were present in both conditions. In both conditions, there are frequent references to the dynamic nature of the experience (moving, fast, change). Words in the Ganzfeld tended to be associated with more uncertainty (faint, shadows, slightly, sort, blurry, disappeared) compared to the Ganzflicker (intense, real).

##### Sleepiness and button press measures

In an exploratory analysis, we looked at the relationship between participants’ measures of sleepiness and their perception of how much the button press interfered with their experience. Our rationale for this was that sleepiness may affect proneness to hallucinations—i.e., in hypnagogia^23^, and may vary across the Ganzflicker and Ganzfeld conditions.

Details for this analysis are given in full in the Supplementary Materials—B*utton press interference and sleepiness*. In brief, paired Wilcoxon signed rank tests suggested that participants felt significantly sleepier in Ganzfeld than in Ganzflicker (V = 0, *p* < 0.001, Supplementary Fig. 3A). There was a negative correlation between sleepiness and the total proportional time spent hallucinating (i.e. as people felt more sleepy, they spent less time in total hallucinating) (r_s(36)_ = −0.38, *p* = 0.012; Supplementary Fig. 4). To see whether this factor contributed to button-press differences between Ganzfeld and Ganzflicker, we ran a moderator analysis for our relevant button press measures (See Supplementary Materials—*Further sleepiness analyses*). Though we did not find any evidence that sleepiness significantly contributed to hallucination frequency, there was some evidence to suggest that sleepiness reduced the average duration of hallucination (exp(B) = 0.84, SE = 0.06, T = −6.24, *p* < 0.001) through a three-way interaction between sleepiness, complexity and experimental condition (exp(B) = 0.76, SE = 0.09, T = −2.24, *p* = 0.026). These analyses suggest that the subjective perception of sleepiness acts to reduce the duration, but not frequency, of complex hallucinations in the Ganzfeld (Supplementary Fig. 5).

##### Hallucinatory proneness and age

Advancing age has been correlated with hallucinatory proneness^56–58^, which may be associated with the development of age-related diseases that are paired with hallucinations^56^. To test this in our data, we carried out an exploratory analysis looking at the difference between age and our button press measures.

There was a positive correlation between the number of Ganzfeld hallucinations and age (Supplementary Fig. 6A; r_s(28)=_0.43, *p* = 0.018) and the proportional duration of Ganzfeld hallucinations and age (Supplementary Fig. 6B; r_s(28)=_0.43, *p* = 0.019). There were no significant correlations found between the number, proportional duration or average duration of Ganzflicker hallucinations and age, or the average duration of Ganzfeld hallucinations and age.

## Discussion

Ganzflicker and Ganzfeld are two visual stimulation methods that can generate visual hallucinations in people without pathological neurocognitive functioning or without the use of psychoactive pharmacological substances. Crucially, these techniques differ in the degree of bottom-up visual input they involve, with Ganzflicker involving a stronger degree of visual stimulation. We compared the phenomenology of the hallucinations induced by these two conditions to gain insight into the mechanisms of simple and complex hallucinations and how they vary across the two conditions. Specifically, we proposed three hypotheses: that simple hallucinations are primarily driven by bottom-up inputs (H1), that complex hallucinations are primarily driven by top-down mechanisms (H2) and that Ganzflicker and Ganzfeld share a common underlying mechanism (H3).

As predicted by H1, we found that Ganzflicker elicited more simple hallucinations than Ganzfeld, which we attribute to the strong visual drive that Ganzflicker provides. This is consistent with the notion that simple hallucinations are primarily driven by bottom-up processes. We also found that both experimental conditions elicited substantially more simple hallucinations than complex hallucinations, suggesting that simple hallucinations are easier to induce experimentally.

There is a multitude of evidence to suggest that low-level aberrant noise, excitability, or direct activation of early visual cortices, which may be interpreted as signal, may be responsible for hallucinatory experiences. Patients with Charles Bonnet syndrome present with a build-up of neural activity in early visual cortex prior to the onset of a hallucination^59^, and respond well to inhibitory stimulation of the visual cortices^60^. Psychedelic drugs such as LSD and psilocybin may bring about some of their low-level geometric visual effects from direct activation of 5HTA receptors, which are populous in early visual cortex, causing excitation^22^. Migraine sufferers, who may experience aura and hallucinations, are thought to have a hyperexcitable brain^61^. It is possible that when the combined effects of steady internal activation and transient external stimulation exceed a certain threshold, pattern formation can occur^33^. For instance, sub-hallucinatory doses of mescaline, when combined with flicker, result in hallucinations above and beyond those normally seen during flicker^33,62^. One possibility building on this notion is that the inherent neural excitability that is present in many hallucinatory states could also be responsible for the high numbers of simple hallucinations in the Ganzfeld. This inherent neuronal excitability could act synergistically with the external input that Ganzflicker provides to result in the increased observed incidence of simple hallucinations in Ganzflicker compared to the Ganzfeld. It is also possible that neural entrainment—the synchronisation of the brain’s intrinsic oscillations with an external rhythm—played a role in the generation of simple hallucinations in the Ganzflicker condition of our experiment^17^. To disentangle the extent to which the likelihood of simple hallucinations during Ganzflicker or similar flicker-induced stimulation can be attributed to entrainment versus pure bottom-up mechanisms, future studies should compare different frequencies of flicker, and arrhythmic flicker, to Ganzfeld.

With regard to complex hallucinations, our results are consistent with the possibility that these are driven by top-down processes to a larger extent (H2). Though there was a higher frequency of simple hallucinations in Ganzflicker compared to Ganzfeld, this relationship was not observed for complex hallucinations. Instead, complex hallucinations were comparable across the two conditions, accompanied by an interaction between complexity and experimental condition. In other words, there was a higher relative likelihood that an experienced hallucination was complex in the Ganzfeld condition than in Ganzflicker. This strongly suggests that bottom-up activation sweeps alone are insufficient to generate complex hallucinations. Instead, top-down processes likely contribute to complex hallucinations independently of visual stimulation technique, perhaps involving a mechanism more akin to mental imagery^28,29,63^. This is in line with a large body of literature that suggests that hallucinations can be elicited through expectations^26,64–66^. Our finding adds to this literature by suggesting that in the absence of explicit expectations, top-down processing might be paired with more complex phenomenology. Similarly, there is a possibility that individual differences in phenomenological control^67,68^, or susceptibility to demand characteristics, could contribute to the generation of specifically complex hallucinations. Future studies should explore the role of demand characteristics (i.e. using the Phenomenological Control Scale^69^) and expectations in the generation of pseudo-hallucinations across Ganzflicker and Ganzfeld.

Despite these differences in simple and complex hallucinations across the Ganzflicker and Ganzfeld conditions, we found a positive correlation between the frequency and proportional duration of hallucinations across these two conditions, as measured by button presses in our paradigm. This was replicated via retrospective questionnaire (the IEQ) scores between the two conditions. This suggests a shared mechanism contributing to Ganzflicker- and Ganzfeld-induced visual hallucinations, despite their differences in stimulation methods and differences in the balance of simple versus complex phenomenology, providing support for H3 and subsequently, prediction 3b (a partially shared mechanism between the two techniques). It is possible that this reflects individual differences in neural excitability which are also linked to pathological states. Our study was not sufficiently powered to disentangle whether the correlation across conditions was driven by simple or complex hallucinations. Therefore, an interesting question for future work is whether this correlation is driven by bottom-up excitability, imagery-like top-down processes, or both.

We also found that Ganzfeld hallucinations were longer compared to Ganzflicker hallucinations. We had no a priori hypotheses about hallucination length, but we speculate that the temporal modulatory input that Ganzflicker provides may contribute to the instability of the hallucinatory state, with each temporal modulation acting as not only a stimulus to pattern formation, but also to disrupt previously elicited patterns^34^. The absence of these disruptions in the Ganzfeld may promote longer hallucinations. We also find that self-reported sleepiness acts to reduce the average length of hallucinations—in particular, complex hallucinations in the Ganzfeld.

A deeper explorative analysis of hallucination content as indicated by words used to describe the experience and associated drawings, revealed a greater presence of form constants in Ganzflicker compared to Ganzfeld. During Ganzflicker, verbal descriptions and drawings often contain references to potential Klüver-like forms such as ‘spirals’, ‘tunnels’, ‘patterns’ and ‘spinning’. Surprisingly, these references were absent in the Ganzfeld condition, raising the possibility that the percepts seen during Ganzfeld arising from internal excitation may lack the activity required to elicit form constants, and thus, bear a closer resemblance to phosphenes. It is also worth considering the relative uncertainty associated with the words used to describe the Ganzfeld condition (for instance shadows, faded, disappeared, slightly, sort). This may suggest that some percepts in Ganzfeld lack the clarity, spatial resolution, and vividness observed in the relatively bottom-up driven Ganzflicker hallucinations^14,27,63,70^. Finally, we also found a positive correlation between age and button-press measures of Ganzfeld hallucinations. Advancing age has been correlated with hallucinatory proneness^56–58^, which may reflect age-related upweighting of predictions about the world^64,65,71^.

Our measures of hallucination frequency and duration rely on button presses. Although these methods provide a quantifiable measure of hallucination onset/offset, they might be influenced by factors not directly related to phenomenology, such as the criterion for when to press. Furthermore, the classification of hallucinations indicated by button presses and prompts as simple or complex relied on subjective judgments by the researchers. Therefore, a crucial aspect of our study was that the results from these button presses and associated responses were replicated by independent retrospective questionnaire measures. This, alongside the absence of correlations between button press measures and participants’ perception of button press interference, validates our approach for quantifying hallucinatory experience. It is important to highlight that the IEQ, a newly developed questionnaire, has not yet undergone validation—additional investigation will be required to ensure its applicability beyond the realm of psychedelic research. However, the consistency and correlation of findings between the ASC and the IEQ offers some initial support for the IEQ's validity in the context of experimentally induced hallucination research.

Our study represents an initial investigation into these mechanisms. Ultimately, self-report measures are, by nature, subjective, difficult to verify and can be influenced by various factors, and this is a major challenge in the study of hallucinations. We further acknowledge that computerised tasks such as the eyes-open Ganzflicker may not be as luminant or as immersive as other methods of flicker light stimulation, which use very powerful, high luminance stroboscopic lamps over closed eyes and may produce a higher frequency of complex hallucinations^45^. In future, the use of additional objective measures, such as neuroimaging techniques, could provide more objective and detailed information about the neural correlates of hallucinations and their phenomenology induced by different stimulation methods. It would also be valuable to further explore the relationship between baseline behavioural measures (such as mental imagery^19,20^ and positive schizotypy^46^) and the propensity to experience complex hallucinations across various stimulation methods. Understanding how individual differences influence hallucination generation could contribute to personalised approaches in hallucination research and potentially inform clinical interventions for individuals experiencing pathological hallucinations.

In conclusion, this study provides insights into the phenomenology of hallucinatory experiences and their relationship with different stimulation techniques. We find evidence to suggest that simple hallucinations in Ganzflicker and Ganzfeld are driven by a bottom-up mechanism, while complex hallucinations more heavily rely on top-down mechanisms which may act independently of visual stimulation techniques.

### Supplementary Information

Supplementary Information.

## Data Availability

Data is available on request. For data requests, please contact the corresponding author (oris.shenyan.15@ucl.ac.uk).
