# Supplementary material for: Visual hallucinations induced by Ganzflicker and Ganzfeld differ in frequency, complexity, and content
Source: Sci Rep. 2024 Jan 29;14:2353. doi: 10.1038/s41598-024-52372-1 (PMC10825158; doi:10.1038/s41598-024-52372-1)
Supplement: Supplementary file 1 — Supplementary Information. [file 41598_2024_52372_MOESM1_ESM.docx]

Visual hallucinations induced by Ganzflicker and Ganzfeld differ in frequency, complexity, and content – Supplementary Materials

Oris Shenyan, Matteo Lisi, John A. Greenwood, Jeremy I. Skipper & Tessa M. Dekker

# Methods

## Questionnaires

The questions comprising the ASC-R can be analysed by dissecting the components into five primary dimensions (5D-ASC - Auditory Alteration, Dread of Ego Dissolution; Oceanic Boundlessness; Reduction of Vigilance; Visionary Restructuralisation) or 11 primary dimensions (11D-ASC - Anxiety; Audio-Visual Synaesthesia; Blissful State; Changed Meaning of Percepts; Complex Imagery; Disembodiment; Elementary Imagery; Experience of Unity; Impaired Control and Cognition; Insightfulness; Spiritual Experience). Specifically, we used questions from the 11D components Elementary Imagery (items 14 and 22). We did not use item 33 from the Elementary Imagery component (I saw lights or flashes of light in total darkness or with closed eyes), due to this being reflected in the nature of the flickering stimuli. We also used questions from the Complex Imagery component (items 39, 72 and 82). Many of these questions were followed by the statement ‘in complete darkness or with closed eyes’; this statement was removed for the purpose of this study as there was always some visual input, and our participants were asked to keep their eyes open. We also utilised one question from the Positive Derealisation subscale of the Oceanic Boundlessness component (5D, item 1) and one question from the visionary reconstruction component (5D, item 7). In addition, a catch question was used (a repetition of item 7) to ensure participants were paying attention to the question.

**Supplementary Table 1**: Items from ASC-R utilised in study

| Dimension | Item | Question |
| --- | --- | --- |
| Elementary Imagery (11D) | 14 | I saw regular patterns [in complete darkness or with closed eyes] |
|  | 22 | I saw colours before me [in total darkness or with closed eyes] |
| Complex Imagery (11D) | 39 | I saw scenes rolling by [in total darkness or with my eyes closed] |
|  | 72 | I could see pictures from my past or fantasy extremely clearly |
|  | 82 | My imagination was extremely vivid |
| Oceanic Boundlessness (5D) | 1 | I felt like I was in a fantastic other world |
| Visionary Reconstruction (5D) | 7 | I saw things that I knew were not real* |

*Question repeated as a catch question. Participants were scored on a percentage difference between a repetition of the catch question (I saw things that I knew were not real) for both hallucinatory conditions. A percentage difference of greater than 30% between the catch and the original question in both conditions was used as criterion for exclusion for the participant. No participants met this criterion.

**Supplementary Table 2:** Items from IEQ utilised in current study

| Dimension | Item | Question |
| --- | --- | --- |
| Complexity | 1 | I saw bursts of light or splashes of colour. |
|  | 2 | I saw abstract geometrical designs and patterns. |
|  | 3 | I saw rapidly transforming objects/ figures. |
|  | 4 | I saw repetitive, moving objects/ figures embedded in geometrical patterns. |
|  | 5 | I saw stable, well-defined objects/ figures. |
|  | 6 | I saw snapshots or glimpses of full scenes |
|  | 7 | I saw full-fledged scenes without being a part of them, similar to watching a movie |
|  | 8 | I was fully immersed within what looked and felt like another authentic realm |
|  | 9 | I was surrounded by a supreme white light |
| Progressive | 17 | My vision progressed over time from simple (busts/splashes/geometries) to complex  (well-defined objects/figures) images. |
|  | 18 | My vision progressed over time from isolated elements to full, immersive scenes. |

*Further experiment details*

Monitoring of, and communication with participants during the Ganzflicker and Ganzfeld sessions of the study took place via video and audio call from a nearby room. Communication was kept to a minimum to avoid interruption of the hallucinatory state. In some cases, the experimenter was present within the room, not in eyesight of the participant. This did not have an effect on any measures collected. After each hallucination condition, retrospective measures of experience (drawings, interviews, and questionnaires) were completed in a separate room.

*Hallucination classification*

**Supplementary Table 3:** Examples of prompts, drawings, and hallucination classification

| Prompt | Drawing | Condition | Hallucination classification |
| --- | --- | --- | --- |
| Spinning kaleidoscope at the bottom, like a tornado | 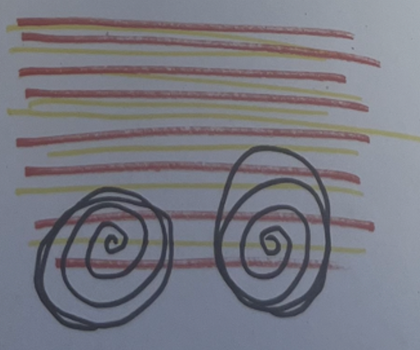 | Ganzflicker | Simple |
| Penguin merged with square shapes | 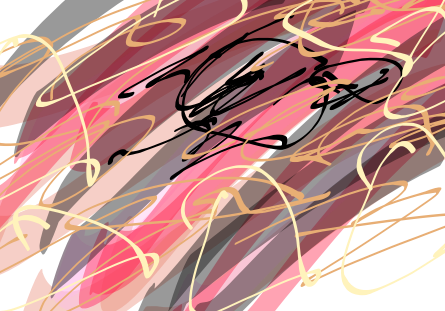 | Ganzflicker | Complex |
| Big wave, dark blue behind it | 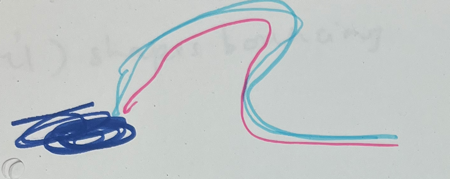 | Ganzflicker | Simple |
| Indistinct dog | 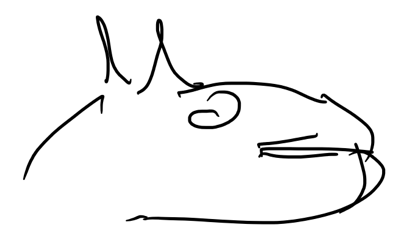 | Ganzflicker | Complex |
| Random shapes | 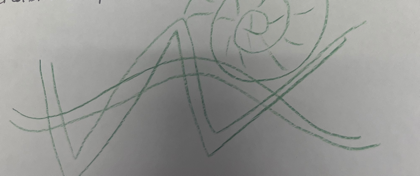 | Ganzflicker | Simple |
| Ring around a planet round a shape diagonally spinning, galaxy | 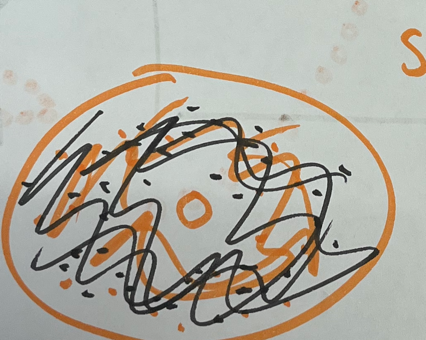 | Ganzflicker | Complex |
| Tunnel goes away like Mario-Kart, rainbow road | 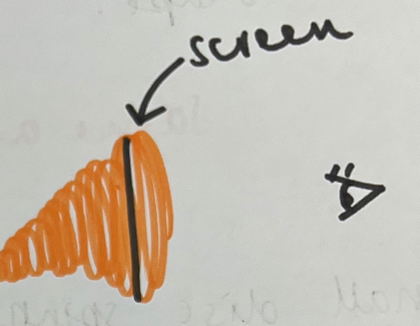 | Ganzflicker | Simple |
| Introduction to Dr Who | 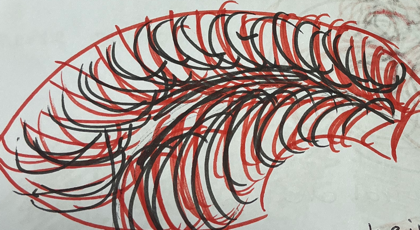 | Ganzflicker | Simple |
| Tortoises | 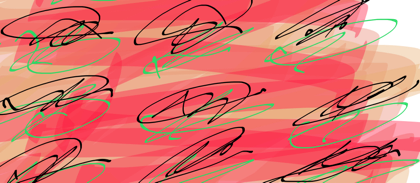 | Ganzflicker | Complex |
| Snowflake | 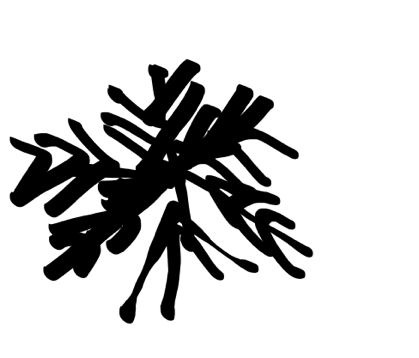 | Ganzflicker | Simple |
| Ball rolling in the middle of the screen | 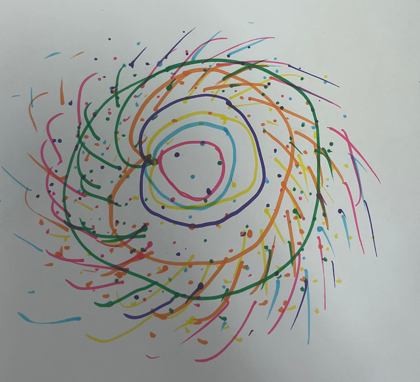 | Ganzflicker | Simple |
| Giant ball on the centre of the screen keeps vibrating | 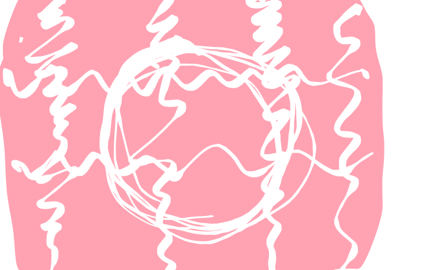 | Ganzflicker | Simple |
| Mirror image of a butterfly | 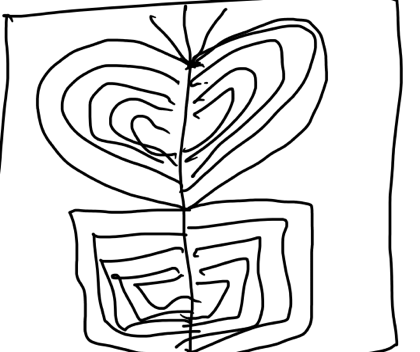 | Ganzflicker | Complex |
| Green electric currents in middle expanding and merging with orange, elements of blue, purple, blue dots | 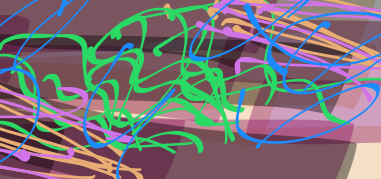 | Ganzflicker | Simple |
| One single caveman face | 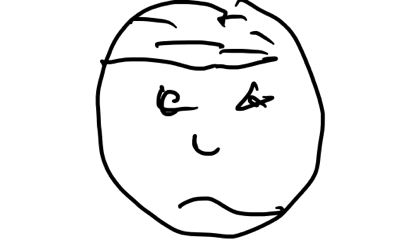 | Ganzflicker | Complex |
| Face | 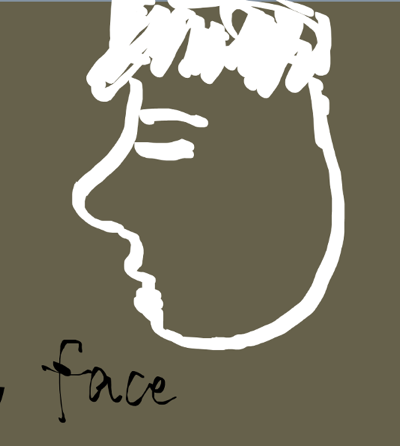 | Ganzfeld | Complex |
| Yellow light shining and growing | 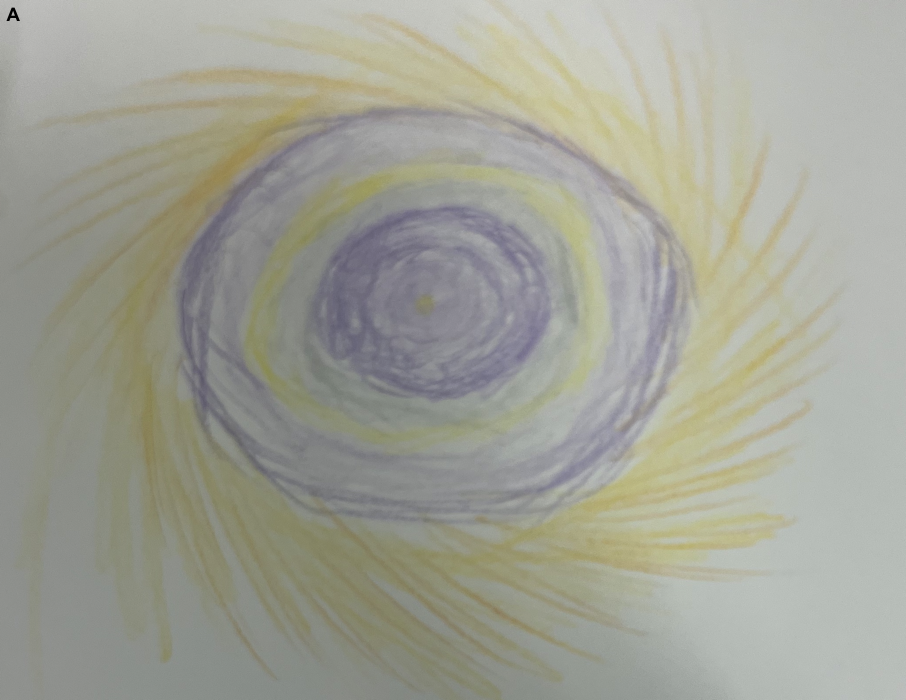 | Ganzfeld | Simple |
| Hollow black and yellow, morphing into the side view of a car, fat controller | 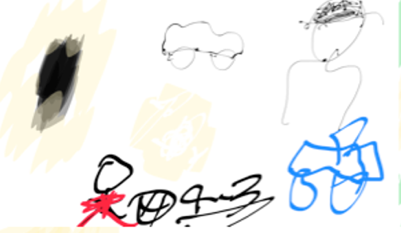 | Ganzfeld | Complex |
| Like polystyrene blistering or burning, filling the visual field | 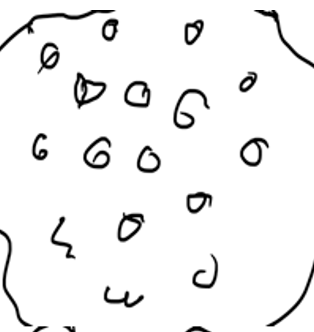 | Ganzfeld | Simple |
| Spiral checkerboard, darker and lighter | 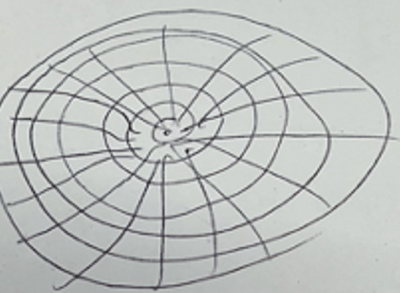 | Ganzfeld | Simple |
| Pulsating mesh | 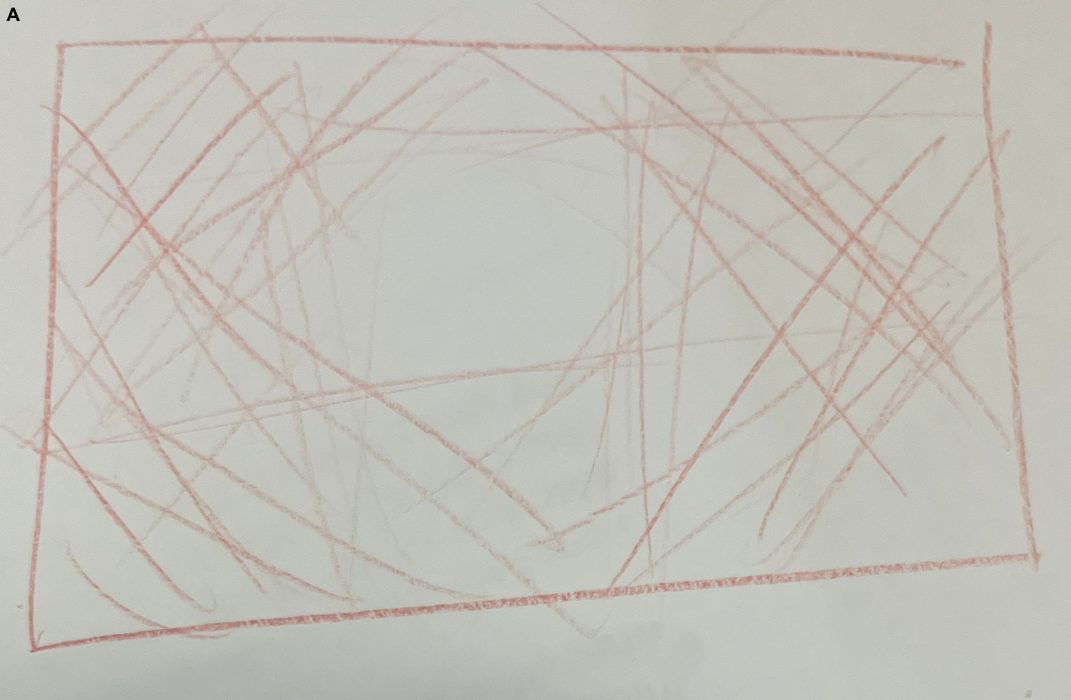 | Ganzfeld | Simple |
| Checked patterns | 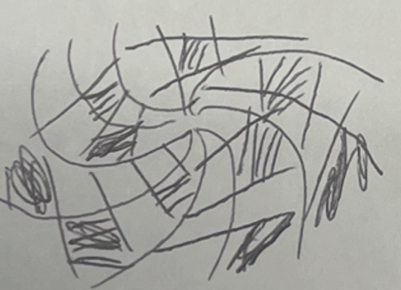 | Ganzfeld | Simple |
| Very faint skull | 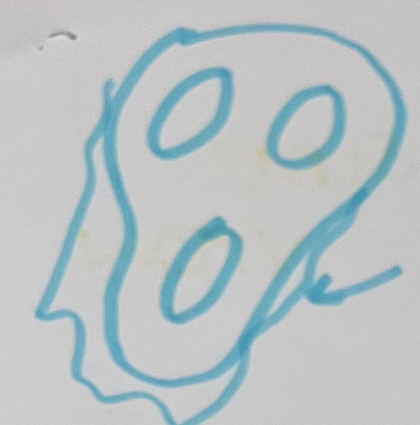 | Ganzfeld | Complex |
| Whispy octopus legs | 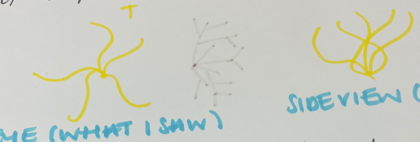 | Ganzfeld | Simple |
| White door | 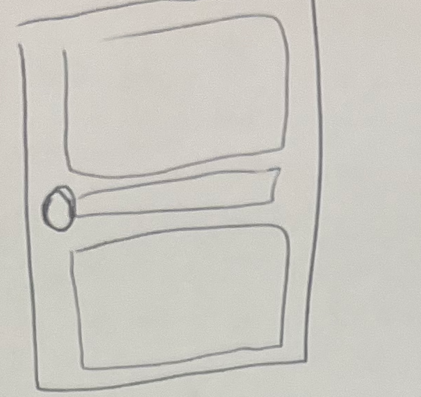 | Ganzfeld | Complex |
| Symmetrical face turning left to right | 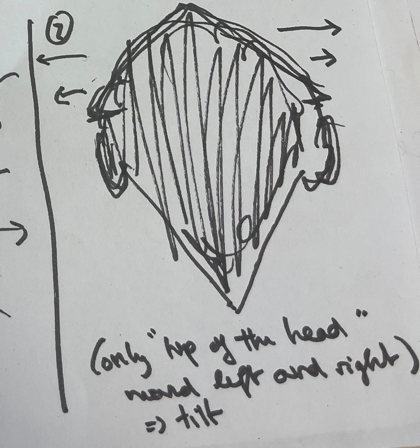 | Ganzfeld | Complex |
| Microbe spots moving into a centre point | 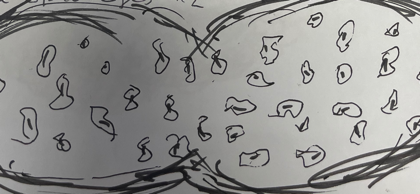 | Ganzfeld | Complex |
| 3D spheres rotating on their axis | 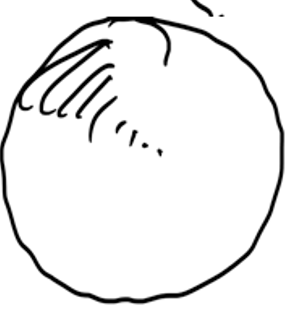 | Ganzfeld | Simple |
| Waves | 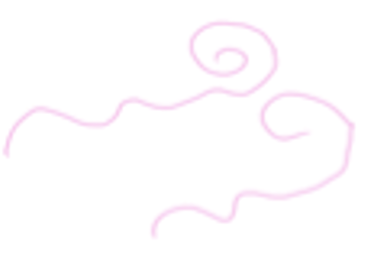 | Ganzfeld | Simple |
| Flashing lines, ovals | 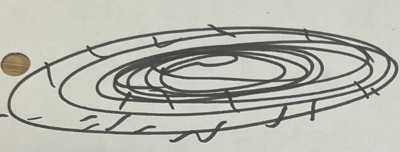 | Ganzfeld | Simple |

*Summary statistics*

**Supplementary Table 4:** Descriptive summary statistics for all relevant quantitative measures. ASC-R – Altered States of Consciousness Rating Scale; IEQ – Imagery Experience Questionnaire; M – mean; SD – standard deviation.

|  | **Ganzflicker** | **Ganzfeld** |
| --- | --- | --- |
|  | *M (SD)* | *M (SD)* |
| Simple hallucinations (number) | 16.17 (14.72) | 5.53 (6.46) |
| Complex hallucinations (number) | 1.40 (2.50) | 1.40 (2.10) |
| Simple hallucinations (duration, seconds) | 11.42 (17.11) | 29.65 (34.93) |
| Complex hallucinations (duration, seconds) | 26.21 (68.00) | 29.14 (45.08) |
| ASC-R Elementary Imagery (0-100) | 65.97 (26.63) | 40.35 (28.75) |
| ASC-R Complex Imagery (0-100) | 24.36 (22.21) | 15.20 (24.61) |
| IEQ Simple Imagery (0-6) | 4.35 (1.54) | 2.24 (1.88) |
| IEQ Complex Imagery (0-6) | 1.27 (1.25) | 0.95 (1.18) |
| Perception of sleepiness (Likert, 1-6) | 1.85 (1.29) | 3.51 (1.47) |
| Perception of interference with button press (Likert, 1-6) | 2.51 (2.03) | 2.45 (2.04) |

# Results

*Bayesian analysis of count data*

**Supplementary Table 5:** *Bayesian negative binomial model analysis of hallucination count. Please note that we report only one parametrization of this model; for our primary analyses within the main text we have estimated similar negative binomial frequentist models by varying the contrast coding of the categorical factors in order to test for all possible contrasts (simple effects). BF – Bayes factor; CI – credible interval; ICC – intercorrelation coefficient;* τ00 *– random intercept variance;* σ2 *– within group variance*

|  | **Log-mean** | **CI (95%)** | **BF_10_** |
| --- | --- | --- | --- |
| **Intercept** | -2.56 | -3.11 – -2.05 | - |
| **Complexity (simple)** | -0.65 | -1.22 – -0.09 | 6.18×10^15^ |
| **Condition (Ganzfeld)** | 2.39 | 1.89 – 2.90 | 3.89 |
| **Interaction** | -0.99 | -1.66 – -0.29 | 15.56 |
| **Random effects** | | | |
| σ2 | 94.53 | Observations: 120  Marginal/Conditional R^2^: 0.250 / 0.648 | |
| τ00 | 49.59 |  |  |
| ICC | 0.67 |  |  |
| N | 30 |  |  |

*Questionnaire validation*


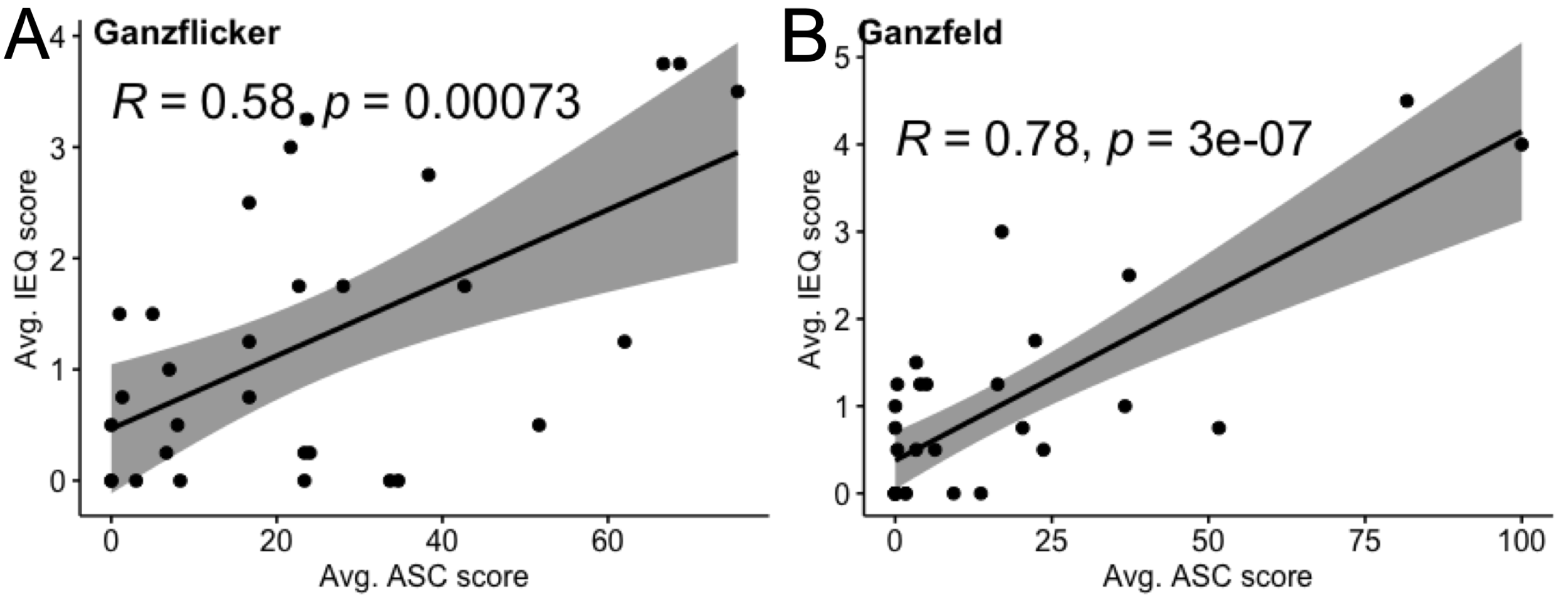


**Supplementary Figure 1:** (A) Scatter plots showing the relationship between average IEQ scores (y-axis) and average ASC scores (x-axis) in Ganzflicker (B) Scatter plots showing the relationship between average IEQ scores (y-axis) and average ASC scores (x-axis) in Ganzfeld. Both plots show associated trend line (black) and Pearson’s correlation correlation testing (95% CI; grey shading) for N=30. ASC – Altered States of Consciousness (rating scale); IEQ – Imagery Experience Questionnaire

*
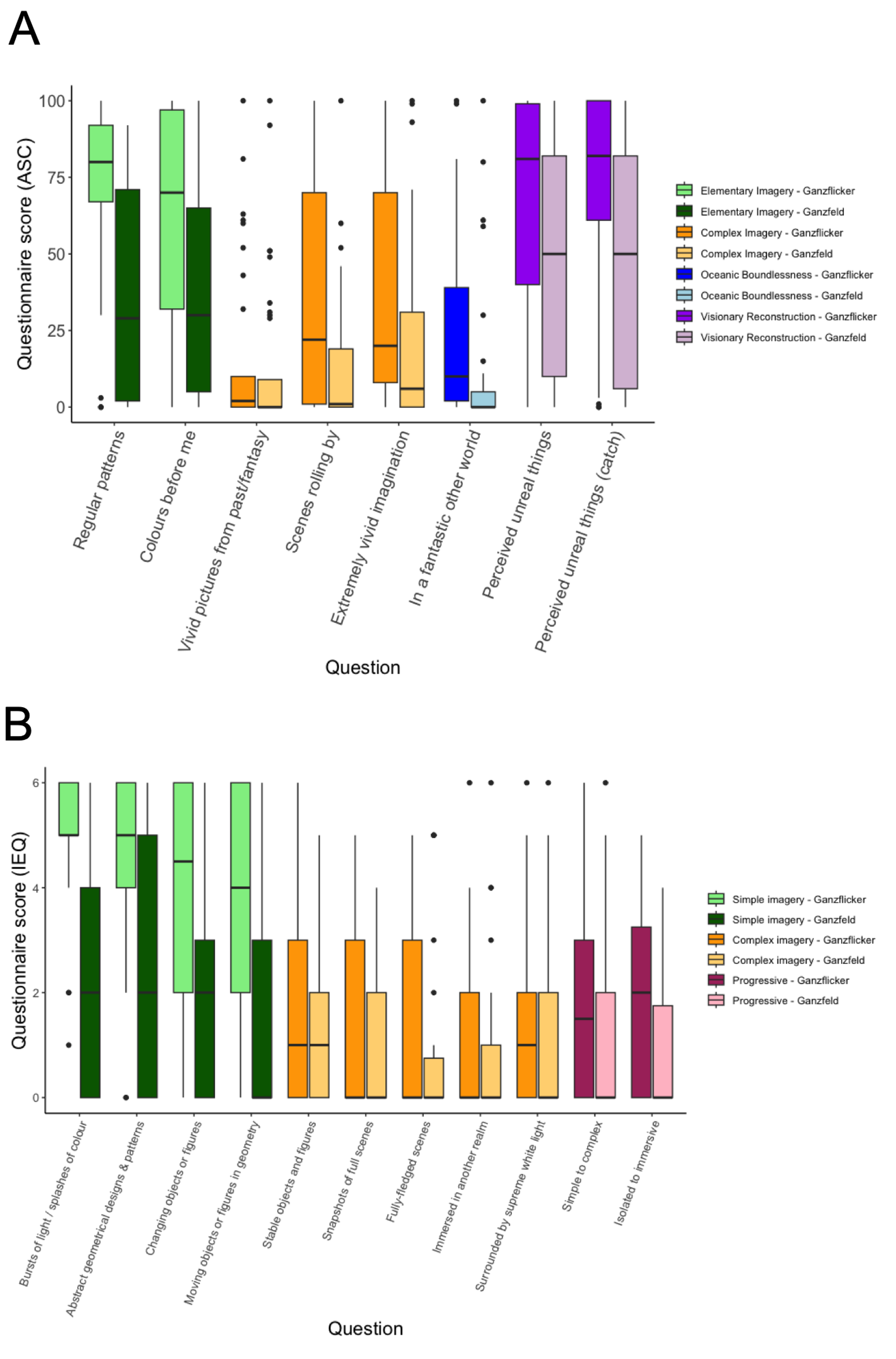
*

**Supplementary Figure 2**: (A) ASC questionnaire results separated by question. Questions are split into separate dimensions (Green – Elementary Imagery; Orange – Complex Imagery; Blue – Oceanic Boundlessness; Purple – Visionary Reconstruction), with the darker shade of each question indicative of Ganzflicker (and the lighter, Ganzfeld), (B) IEQ questionnaire results separated by question. Questions are split into separate dimensions (Green – Simple Imagery; Orange – Complex Imagery; Pink – Progressive Imagery), with the darker shade of each question indicative of Gan`flicker (and the lighter, Ganzfeld). Questions are abbreviated; please see Supplementary Tables 2 and 3 for unabbreviated questions. Both conditions are *N*=30, for both conditions. ASC – Altered States of Consciousness (rating scale); IEQ – Imagery Experience Questionnaire

####

#### Button press interference and sleepiness

Paired Wilcoxon rank signed tests were carried out to see how participants' sleepiness and their perception of how much the button press interfered with their experience varied between Ganzfeld and Ganzflicker. Two participants were removed from both analyses pertaining to sleepiness and those pertaining to button press interference as their data was not recorded for one condition, leaving n=28 (as their data for their other condition was also excluded). Five further participants were excluded from the button press analyses as they had no hallucinations in one condition, therefore they could not provide an opinion on how much they thought the button presses interfered with their experience, resulting in n=23 (as their data for their other condition was also excluded).

Paired Wilcoxon signed rank tests suggested a difference between participants' sleepiness in Ganzflicker compared to Ganzfeld (V=14, p<0.001). There was no difference between how much participants perceived the interference of the button press in either condition (V=74, p=0.77).


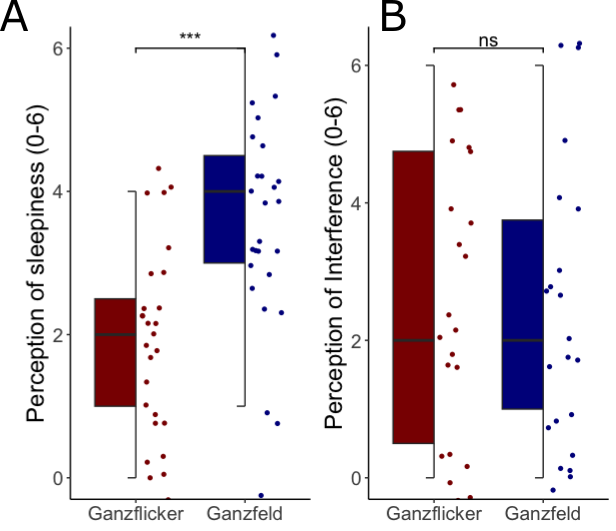


**Supplementary Figure 3:** (A) Relationship between experimental condition (x-axis: red – Ganzflicker; blue – Ganzfeld) and participants perception of sleepiness (y-axis), *N=28* (B) Relationship between experimental condition (x-axis; red – Ganzflicker; blue – Ganzfeld) and participants perception of how much the button press interfered with their hallucinatory experience (y-axis), *N*=23. Statistics shown are significance testing for paired Wilcoxon rank signed tests.

*Word frequency analysis – Open interview data*

**Supplementary Table 6:** Word frequency analysis showing the frequency of words used three or more times by participants in open interviews following Ganzflicker and Ganzfeld

| **Ganzflicker words, n (%)** | **Ganzfeld words, n (%)** |
| --- | --- |
| shape, 36 (3.46%) | shape, 33 (3.92%) |
| moving, 30 (2.89%) | time, 17 (2.02%) |
| pattern, 22 (2.12%) | guess, 16 (1.9%) |
| colour, 20 (1.92%) | sort, 15 (1.78%) |
| circle, 18 (1.73%) | yellow, 13 (1.54%) |
| sort, 18 (1.73%) | colour, 12 (1.43%) |
| lines, 17 (1.64%) | dots, 10 (1.19%) |
| guess, 14 (1.35%) | moving, 9 (1.07%) |
| change, 13 (1.25%) | lines, 9 (1.07%) |
| fast, 13 (1.25%) | circles, 8 (0.95%) |
| blue, 12 (1.15%) | stuff, 8 (0.95%) |
| time, 12 (1.15%) | feel, 7 (0.83%) |
| feel, 12 (1.15%) | front, 7 (0.83%) |
| white, 9 (0.87%) | couldnt, 6 (0.71%) |
| times, 8 (0.77%) | minutes, 6 (0.71%) |
| tunnel, 8 (0.77%) | report, 6 (0.71%) |
| describe, 8 (0.77%) | disappeared, 6 (0.71%) |
| yellow, 6 (0.58%) | faint, 6 (0.71%) |
| cross, 6 (0.58%) | blue, 5 (0.59%) |
| grey, 6 (0.58%) | pattern, 5 (0.59%) |
| stuff, 6 (0.58%) | hard, 5 (0.59%) |
| background, 5 (0.48%) | left, 4 (0.48%) |
| intense, 5 (0.48%) | darker, 4 (0.48%) |
| compared, 5 (0.48%) | shadows, 4 (0.48%) |
| recognise, 5 (0.48%) | faded, 4 (0.48%) |
| focus, 5 (0.48%) | blinked, 4 (0.48%) |
| dots, 4 (0.38%) | blobs, 4 (0.48%) |
| move, 4 (0.38%) | quickly, 4 (0.48%) |
| waves, 4 (0.38%) | tiny, 4 (0.48%) |
| spirals, 4 (0.38%) | majority, 4 (0.48%) |
| complex, 4 (0.38%) | real, 4 (0.48%) |
| hallucinations, 4 (0.38%) | clouds, 4 (0.48%) |
| movement, 4 (0.38%) | created, 4 (0.48%) |
| middle, 4 (0.38%) | stay, 4 (0.48%) |
| road, 4 (0.38%) | larger, 3 (0.36%) |
| cut, 4 (0.38%) | line, 3 (0.36%) |
| constant, 4 (0.38%) | experience, 3 (0.36%) |
| dogs, 4 (0.38%) | blurry, 3 (0.36%) |
| person, 4 (0.38%) | dot, 3 (0.36%) |
| tended, 4 (0.38%) | round, 3 (0.36%) |
| triangles, 4 (0.38%) | duf, 3 (0.36%) |
| head, 4 (0.38%) | eye, 3 (0.36%) |
| dunno, 4 (0.38%) | oval, 3 (0.36%) |
| direction, 3 (0.29%) | press, 3 (0.36%) |
| left, 3 (0.29%) | slightly, 3 (0.36%) |
| line, 3 (0.29%) | distinct, 3 (0.36%) |
| sky, 3 (0.29%) | flashes, 3 (0.36%) |
| bottom, 3 (0.29%) | happen, 3 (0.36%) |
| figure, 3 (0.29%) | head, 3 (0.36%) |
| forming, 3 (0.29%) | move, 3 (0.36%) |
| stars, 3 (0.29%) | waves, 3 (0.36%) |
| bigger, 3 (0.29%) | wouldnt, 3 (0.36%) |
| top, 3 (0.29%) | difficult, 3 (0.36%) |
| watching, 3 (0.29%) | found, 3 (0.36%) |
| grids, 3 (0.29%) | physical, 3 (0.36%) |
| difference, 3 (0.29%) | pressed, 3 (0.36%) |
| static, 3 (0.29%) | lights, 3 (0.36%) |
| lights, 3 (0.29%) | basically, 3 (0.36%) |
| heart, 3 (0.29%) | circle, 3 (0.36%) |
| looked, 3 (0.29%) | tired, 3 (0.36%) |
| specific, 3 (0.29%) | abstract, 3 (0.36%) |
| rainbow, 3 (0.29%) | changed, 3 (0.36%) |
| bang, 3 (0.29%) | werent, 3 (0.36%) |
| happen, 3 (0.29%) | imagining, 3 (0.36%) |
| happening, 3 (0.29%) | ball, 3 (0.36%) |
| scenes, 3 (0.29%) | animals, 3 (0.36%) |
| forms, 3 (0.29%) | periphery, 3 (0.36%) |
| illusions, 3 (0.29%) | mind, 3 (0.36%) |
| press, 3 (0.29%) | noise, 3 (0.36%) |
| pressed, 3 (0.29%) | thinking, 3 (0.36%) |
| imagination, 3 (0.29%) | darkness, 3 (0.36%) |
| real, 3 (0.29%) | fade, 3 (0.36%) |
| tired, 3 (0.29%) | perception, 3 (0.36%) |
| uncomfortable, 3 (0.29%) | create, 3 (0.36%) |
| couldnt, 3 (0.29%) | relaxing, 3 (0.36%) |
| concrete, 3 (0.29%) | - |
| minutes, 3 (0.29%) | - |
| quickly, 3 (0.29%) | - |
| abstract, 3 (0.29%) | - |
| difficult, 3 (0.29%) | - |
| people, 3 (0.29%) | - |
| pretty, 3 (0.29%) | - |
| remember, 3 (0.29%) | - |
| report, 3 (0.29%) | - |
| flickering, 3 (0.29%) | - |
| reason, 3 (0.29%) | - |
| structures, 3 (0.29%) | - |
| lots, 3 (0.29%) | - |
| objects, 3 (0.29%) | - |
| blink, 3 (0.29%) | - |
| relaxed, 3 (0.29%) | - |
| faint, 3 (0.29%) | - |

*Further sleepiness analyses*


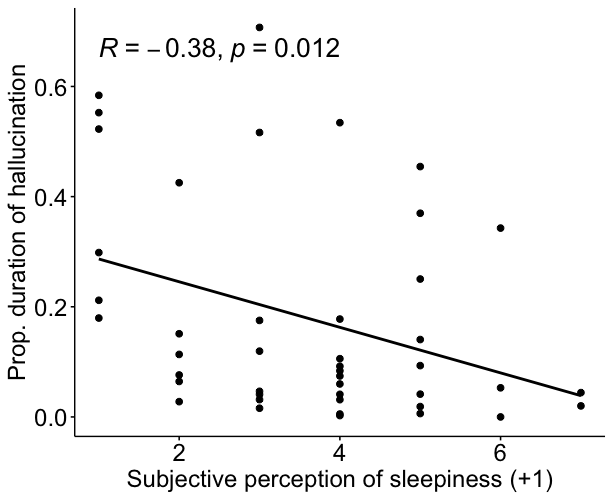


**Supplementary Figure 4:** Relationship between participants subjective perception of sleepiness (0-6 +1 for analysis and visualisation purposes) (x-axis) and proportional total time spent hallucinating across both experiments (y-axis), *N*=28. Spearman’s rank correlation and associated p-value shown on plot.

As a follow-up to the significant correlation between participants' self-reported sleepiness and their total proportional time spent hallucinating (Supplementary Figure 4), we carried out a moderator analysis to examine the influence of perceived sleepiness on our key button press measures, specifically hallucination frequency and average duration of hallucinations. We built a model including all main effects and interactions (experimental condition, sleepiness and hallucination complexity) for our key button-press outcomes (hallucination frequency and average hallucination duration).


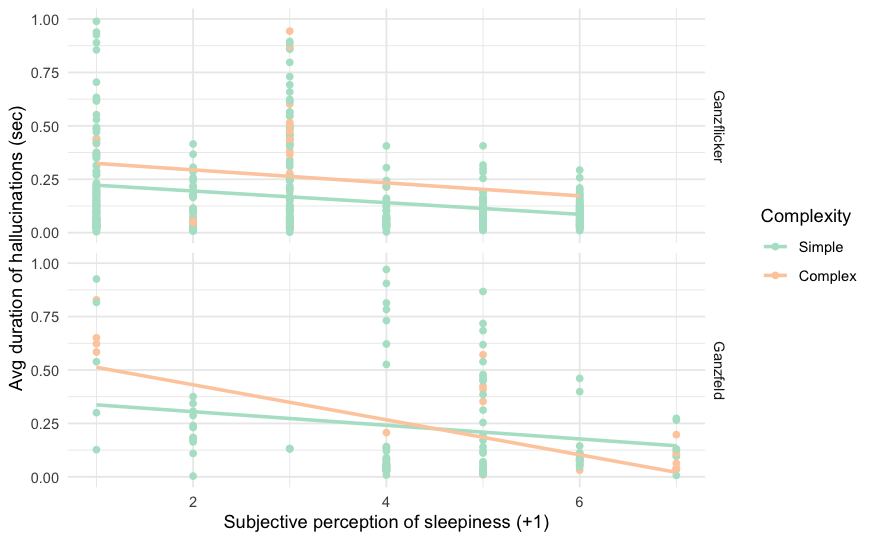


**Supplementary Figure 5:** (A) Scatter plots showing the relationship between the average duration of discrete hallucinatory periods (y-axis) and subjective perception of sleepiness (0-6 +1 for analysis and visualisation purposes; x-axis) for Ganzflicker (top) and Ganzfeld (bottom) split out by hallucination complexity (green - simple; orange - complex). Both plots show associated trend lines. *N*=28.

*Age*


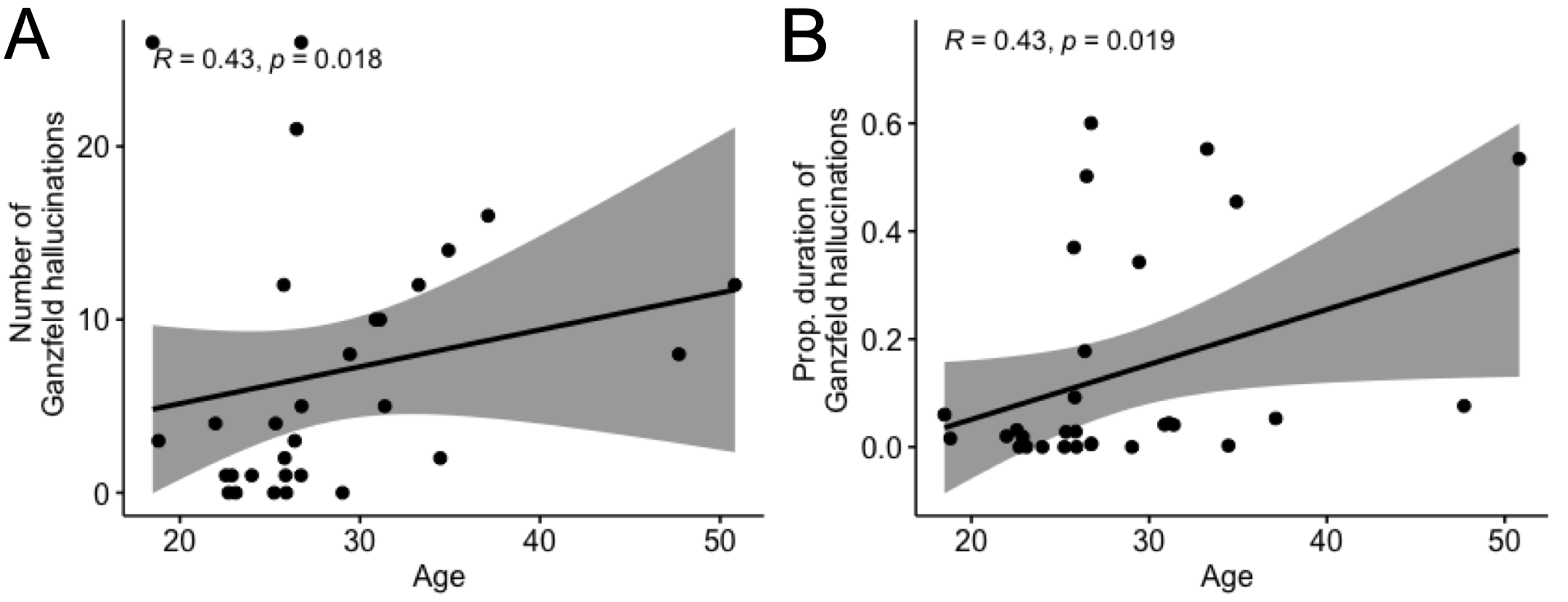


**Supplementary Figure 6:** (A) Scatter plots showing the relationship between the number of Ganzfeld hallucinations (y-axis) and age (x-axis) (B) Scatter plot showing the relationship between the total proportional time spent hallucinating during Ganzfeld (y-axis) and age (x-axis). Both plots show associated trend line (black) and Spearman’s rank correlation testing (95% CI; grey shading) for *N*=30.
